# Comparative study on comprehensive quality of Xinhui chenpi by two main plant propagation techniques

**DOI:** 10.1002/fsn3.3148

**Published:** 2022-11-20

**Authors:** E‐yu Tan, Fang Li, Xinheng Lin, Shaofeng Ma, Guanghua Zhang, Hua Zhou, Yue Ouyang, Ziyu Tang, Qiqing Cheng

**Affiliations:** ^1^ School of Pharmaceutical Science, Guangzhou University of Chinese Medicine Guangzhou People's Republic of China; ^2^ Jiangmen Wuyi Hospital of Traditional Chinese Medicine Jiangmen People's Republic of China; ^3^ Guangdong Provincial Hospital of Chinese Medicine Guangdong Provincial Academy of Chinese Medical Sciences, State Key Laboratory of Dampness Syndrome of Chinese Medicine Guangzhou People's Republic of China; ^4^ Second Affiliated Hospital of Guangzhou University of Chinese Medicine Guangdong‐Hong Kong‐Macau Joint Lab on Chinese Medicine and Immune Disease Research Guangzhou People's Republic of China; ^5^ Faculty of Chinese Medicine and State Key Laboratory of Quality Research in Chinese Medicine Macau University of Science and Technology Taipa People's Republic of China

**Keywords:** *Citrus limonia*, *Citrus reticulata* “Chachi”, cutting, grafting, quality, Xinhui chenpi

## Abstract

Xinhui chenpi (XHCP), the sun‐dried peel of the mandarin orange, *Citrus reticulata* “Chachi,” is the most famous crude drug, as well as a traditional seasoning in Chinese cooking. The main cultivation methods of XHCP are cutting and grafting, but it is generally considered that the quality of XHCP after cutting is superior to that obtained from plants propagated by graftings, which had a negative impact on the marketing of the finished product. In our study, a total of 25 samples of XHCP obtained from plants cultivated by either traditional methods (i.e., from cuttings) or by grafting were collected to compare the contents of four types of metabolites (essential oils, flavonoids, synephrine, and total polysaccharides) as well as antioxidant activity. The results revealed that the quality of XHCP did not decline after cutting, and marked individual differences between XHCP samples, even when prepared from plants grown in the same way. In general, grafting had no significant effect on the most essential oils components, total polysaccharides, synephrine, total flavonoids, total polymethoxylated flavones, hesperidin, nobiletin, tangeretin content, and antioxidant activity. Nevertheless, five volatile compounds can be used as potential chemical markers (*p* < 0.05) to distinguish between cutting XHCP and grafted XHCP, while four volatile compounds showed high content in grafted XHCP. Our study is expected to provide a theoretical basis for XHCP breeding and cultivation, and thereby further standardize the market of XHCP.

## Graphical abstract

There was no significant difference in the content of flavonoids, polysaccharides, synephrine and antioxidant activity between grafted and cutting Citrus dried peels;Five volatile components were significantly different between grafted and cutting *Citrus* dried peels, among which four were significantly higher in grafted peel;The five significantly different volatile components can be used as potential chemical markers to distinguish cutting and grafted peels;

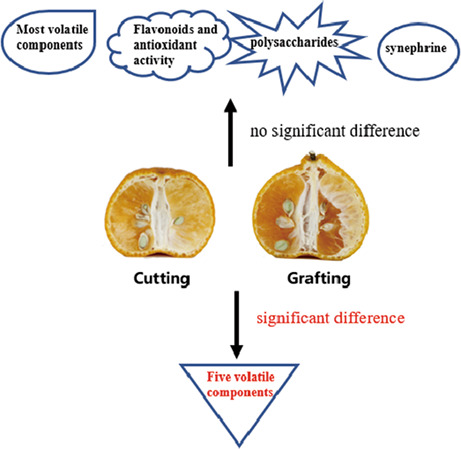

## 1 INTRODUCTION

Xinhui chenpi (XHCP), derived from the dried mature peels of *Citrus reticulata* “Chachi,” is used as traditional Chinese medicine (chenpi is listed in the Chinese Pharmacopoeia Commission, 2020) and as a food seasoning in Chinese cuisine. Its role as a Chinese herbal medicine has been widely studied (Lai, 2018; Lu, 1999; Zhang, 2007), with authentic medicinal forms of XHCP being produced in Xinhui Jiangmen (Guangdong Province, China).

XHCP has brand value and considerable market potential as one of the most important industries in Jiangmen. Thus, the overall production value of the XHCP industry was RMB 1.6 billion in 2015, increasing to RMB 6.6 billion by 2018, and exceeding 10 billion CNY in 2021 (The paper, 2022).

**FIGURE 1:**
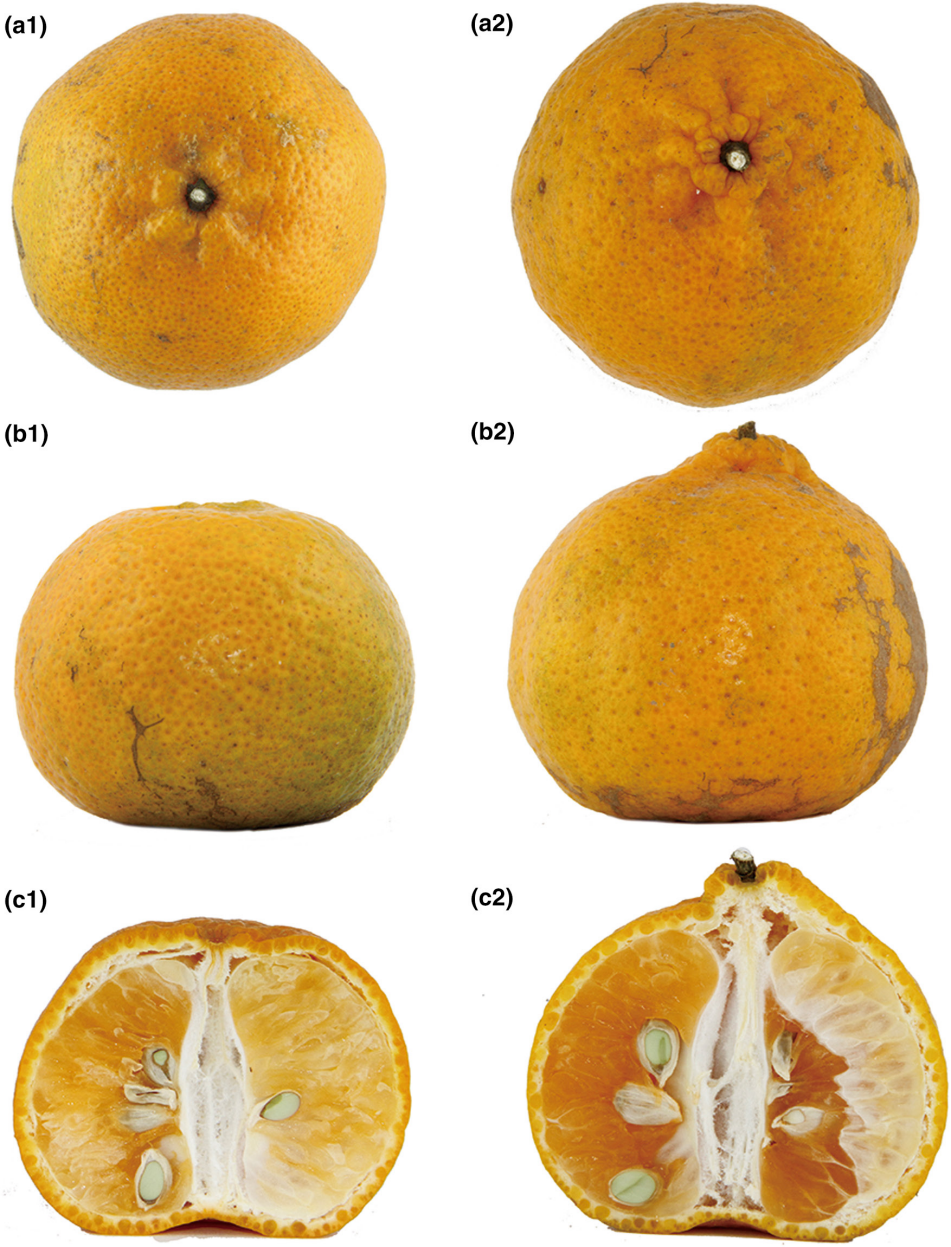
The appearance of fruits obtained by the two planting methods. (1 for cutting; 2 for grafting; (a) for plans; (b) for elevations, and (c) for longitudinal section)

However, Xinhui *C. reticulata* shows weak disease resistance to citrus huanglongbing, also known as citrus greening disease, and low yield, which has caused problems in meeting the rapidly growing demand of the market (Huang, 1929; Jian, 2009). To address the aforesaid deficiencies, *C. limonia* (rangpur) rootstock was introduced for the grafting of *C. reticulata* in Xinhui in the mid‐1950s. In the ensuing decades, this rootstock has proved ideal for the production of Xinhui citrus and has been adopted by most growers (Huang, 1929; Jian, 2009). Nevertheless, it is generally considered that the quality of XHCP after grafting is inferior to that obtained from plants propagated by cuttings (e.g., the obvious differences in the appearance of fruits obtained by the two planting methods) (Figure 1). Therefore, some growers continue to cultivate Xinhui citrus saplings from cuttings. The high profile of XHCP produced in this way, combined with relatively poor yields, resulted in an elevated sales price for the finished product.

The rapid development of the market for XHCP and the increased awareness of the importance of genuine medicinal materials have led to some controversy over the decline in the quality of XHCP after grafting. There have been some recent reports on the effect of rootstocks on fruit quality, but results vary (Aguilar‐Hernández et al., 2020; Antonella et al., 2003; Cardeñosa et al., 2015; Feng et al., 2018; Machado et al., 2017; Morales et al., 2021; Raddatz‐Mota et al., 2019). However, studies that compare the quality of XHCP produced from plants produced by grafting or from cuttings are lacking. Accordingly, we aimed here to qualitatively and quantitatively analyze the four types of metabolites (essential oils, flavonoids, synephrine, and total polysaccharides) of peel from both types of *C. reticulata* plant, and to compare their antioxidant activities, to comprehensively determine any quality differences. Our study is expected to provide a theoretical basis for XHCP breeding and cultivation, and thereby scientific guidance for further studies into the quality of XHCP.

## 2 MATERIALS AND METHODS

### 2.1 Materials

Cutting XHCP samples were gathered from 13 *Citrus* plantations, while grafted XHCP samples were collected from another 12 *Citrus* plantations (Table 1). When gathering samples, it is ensured that the grafting mode of the trees and the development stage of fruit were the same. After gathering, the peels were taken and sun‐dried as experimental samples. All samples were labeled according to their source information and authenticated by the authors, then powdered using a mill. A sieve (0.425 mm) was used to homogenize the powder.

**TABLE 1:** Collecting information on samples.

Sample	Cutting or grafting	Production areas (Jiangmen, Guangdong, China)
C1	C	Meijiang
C2	C	Chakeng
C3	C	Fengcheng
C4	C	Jiuzisha
C5	C	Huichun
C6	C	Wubao
C7	C	Yuchong
C8	C	Dongjia
C9	C	Jiuzishahenan
C10	C	Nantan
C11	C	Shuangshui
C12	C	Tianma
C13	C	Guantian
G1	G	Huicheng
G2	G	Shenlv
G3	G	Meijiang
G4	G	Wubao
G5	G	Yaxi
G6	G	Sanjiang
G7	G	Gujin
G8	G	Nantan
G9	G	Qibao
G10	G	Guantian
G11	G	Shuangshui
G12	G	Qihaotang

All reference materials used were purchased from Manster (Chengdu, China). The reagents and solvents used for GC–MS and HPLC were purchased from Macklin (Shanghai, China) of HPLC purity. The remaining reagents were supplied by Damao (Tianjin, China).

### 2.2 Gas chromatography–mass spectrometry

The extraction of essential oils was referred to the Chinese Pharmacopeia (Chinese Pharmacopoeia Commission, 2020). GC–MS data were acquired using a GC–MS‐QP2020 NX system (Shimadzu, Japan). Separation was achieved using an SH‐Rxi‐5Sil capillary column (30 m × 0.25 mm, 0.25 μm film thickness; Shimadzu, Japan). The oven temperature was initially set at 60°C (0 min hold), then ramped at 1°C/min to 80°C for 2 min, and finally, increased at 5°C/min to 180°C for 4 min. The carrier gas for this analysis was helium, which was maintained at a flow rate of 1 ml/min. The injection volume was 1 μl with a split ratio of 20:1. The temperature of the GC injector was set at 270°C. Electron impact mode (EI) ion sources were used for mass spectra over a scan range 30–550 m/z. Volatile compounds were identified using the NIST library. The relative percentage of volatile oil constituents was determined by normalization from the GC peak areas.

### 2.3 Ultraviolet spectrophotometry

The content of total flavonoids, total polymethoxylated flavones, and total polysaccharides was determined by UV spectrophotometry. Total flavonoid content was determined using a zirconium oxychloride colorimetric method according to Wang et al. (2013) and Wei et al. (2013), while total polymethoxylated flavones content was measured as described by Luo et al. (2017). Total polysaccharides content was determined using the phenol–sulfuric acid method under the Chinese Pharmacopeia (Chinese Pharmacopoeia Commission, 2020).

### 2.4 High‐performance liquid chromatography

HPLC data were acquired using a 1260 Infinity II LC system (Agilent Technologies, Waldbronn, Germany). The determination of three active flavonoids (hesperidin, nobiletin, and tangeretin) and one alkaloid (synephrine) was performed as described by Zheng et al. (2019). The four compounds were quantified using the corresponding reference standards.

### 2.5 Method validation

**Linearity** To ensure that each assay was used within a linear range, a calibration curve of reference standards was constructed using the analyte concentration and the area of the corresponding peak on the chromatogram.

**Reproducibility** Sample solutions were assessed six times to evaluate the reproducibility of each method and the relative standard deviation (*RSD*) of each compound was calculated.

**Stability** Each sample solution was injected six different times within 24 h and the *RSD* value was calculated to evaluate the stability.

**Accuracy** The accuracy of the method was determined by analyzing the recovery percentage of the constituents to be quantified in the test solution. The recovery percentage was determined by comparing the increased peak area, after the addition of the sample to a fixed amount of reference substance, to the theoretical peak area of the fixed amount of reference substance.

### 2.6 Antioxidant activity

**DPPH** The DPPH assay was adapted from the method of Pang et al. (2017). A_0_: The reference solution was obtained by mixing 2 ml DPPH solution with 1 ml methanol; the absorbance was determined at 519 nm after a 30‐min incubation period at room temperature in the dark. A_1_: Appropriately diluted sample extracts (made up to 1 ml with methanol) were added to 2 ml ethanol. After 30 min incubation at room temperature in the dark, the absorbance at 519 nm was measured. A_2_: Appropriately diluted sample extracts (made up to 1 ml with methanol) were added to 2 ml DPPH solution. After 30 min incubation at room temperature in the dark, the absorbance was measured at 519 nm. The DPPH scavenging activity was calculated using the formula, DPPH% = (1‐(A_2_‐A_1_)/A_0_) × 100%.

**FRAP** The FRAP assay was adapted from the method of Liew et al. (2018). FRAP reagent consists of a solution containing 10 mM 2,4,6‐tripyridyl‐*S*‐triazine, 20 mM FeCl_3_, and 300 mM acetate buffer (pH 3.6) at a ratio of 1:1:10. To 0.3 ml sample extract or standard solution, 2.7 ml preheated FRAP reagent was added and incubated in the dark at 37°C for 5 min, then the absorbance was measured at 593 nm. Standard solutions of FeSO_4_ at five different concentrations (25–400 μM) were used to construct the calibration curve. The antioxidant capacity of each sample was expressed according to the equivalent concentration of FeSO_4_ standard solution (μmol/L).

### 2.7 Statistical analysis and OPLS‐DA analysis

Experimental data that showed both normal distribution and homogeneous variance were compared by one‐way analysis of variance (ANOVA) followed by a post‐hoc LSD test for multiple comparisons. Otherwise, a nonparametric test (Mann–Whitney U) was used. To further investigate the four types of metabolites differences between cutting and grifting XHCP and potential biomarkers, orthogonal partial least squares discriminant analysis (OPLS‐DA) was subsequently performed using SIMCA software (Version 14.1).

## 3 RESULTS

### 3.1 Method validation

Good linear behavior was observed for all six components studied, with a regression coefficient (R^2^) above 0.999 (Table S1). The RSD of reproducibility of the six chemical compositions (total flavonoids, total polymethoxylated flavones, hesperidin, nobiletin, tangeretin, synephrine, and total polysaccharides) varied from 0.15% to 2.74%, while the RSD of stability ranged from 0.64% to 2.13% (Table S2). The recovery values were between 95.17% and 104.02% (Table S2). The above results indicate that the proposed methods can be used to determine the selected analyte in XHCP with high reproducibility, stability, and accuracy.

### 3.2 Effect of cutting and grafting on volatile components

The volatile components detected in each of the 25 samples were highly variable, ranging from 30 to 62 in number, with a total of 108 components identified (Table S3, Table S4, Table S5, Table S6, Figure S1). Twenty volatile components were present in all cutting and grafted groups, including D‐Limonene, γ‐Terpinene, β‐Myrcene, and Benzoic acid, 2‐(methylamino)‐, methyl ester, and together these accounted for a relatively large percentage of the total components. The highest number of essential oil components was detected in G2 (i.e., grafted sample 2), followed by G11. In general, though, there was no statistically significant difference in the number of different essential oils detected in the cutting and grafted groups (*p =* .737 > .05). A statistical analysis found significant differences in the content of α‐Thujene, α‐Pinene, Sabinen, β‐Pinene, and α‐Sinensal between the two groups (Table 2); however, only for α‐Sinensal was the content higher in cutting XHCP than in grafted XHCP.

**TABLE 2:** Statistical analysis (*n* = 3)

	Cutting	Grafting	*p*‐value
α‐Thujene	0.38%	0.60%	<.001
α‐Pinene	1.30%	2.15%	<.001
Sabinen	0.18%	0.23%	.002
β‐Pinene	1.19%	1.67%	<.001
β‐Myrcene	2.17%	2.32%	.088
α‐Phellandrene	0.06%	0.053%	.979
o‐Cymene	3.24%	3.45%	.611
D‐Limonene	69.77%	68.03%	.470
γ‐Terpinene	15.68%	14.67%	.168
Terpinolene	0.93%	0.81%	.077
Linalool	0.13%	0.19%	.295
4‐Terpineol	0.17%	0.25%	.098
α‐Terpineol	0.34%	0.50%	.168
Decanal	0.25%	0.32%	.936
Perillaldehyde	0.06%	0.07%	.574
Benzoic acid, 2‐(methylamino)‐, methyl ester	1.23%	1.38%	.497
Caryophyllene	0.15%	0.13%	.152
α‐Selinene	0.05%	0.05%	.295
α‐Farnesene	0.77%	0.67%	.538
α‐Sinensal	0.73%	0.52%	.030
Total polysaccharides	67.48 mg/g	66.66 mg/g	.871
Synephrine	1.94 mg/g	2.15 mg/g	.077
Total flavonoids	59.66 mg/g	61.07 mg/g	.631
Total polymethoxy flavonoids	7.55 mg/g	7.92 mg/g	.467
Hesperidin	30.78 mg/g	33.39 mg/g	.205
Nobiletin	3.52 mg/g	3.33 mg/g	.370
Tangeretin	2.53 mg/g	2.52 mg/g	.968
Antioxidant activity in DPPH	73.50%	71.14%	.292
Antioxidant activity in FRAP	634.01 μmol/L	656.26 μmol/L	.305

### 3.3 Quantitative analysis of total polysaccharides and synephrine in cutting and grafted XHCP

The content of total polysaccharides varied greatly within the groups: in cutting XHCP, the total polysaccharides content ranged between 47.82 and 97.79 mg/g, while in grafted XHCP, it varied from 54.86 to 91.77 mg/g (Table S7). The content of the synephrine was rather lower: in cutting XHCP, synephrine content ranged from 1.54 to 2.73 mg/g, while in grafted XHCP, the content was between 1.72 and 2.41 mg/g (Table S7). Statistical analysis showed that the propagation method had no significant effect on the content of total polysaccharides and synephrine (*p* = .871 > .05, *p* = .077 >.05) (Table 2).

### 3.4 Effect of cutting and grafting on the flavonoid content and antioxidant activity

**FIGURE 2:**
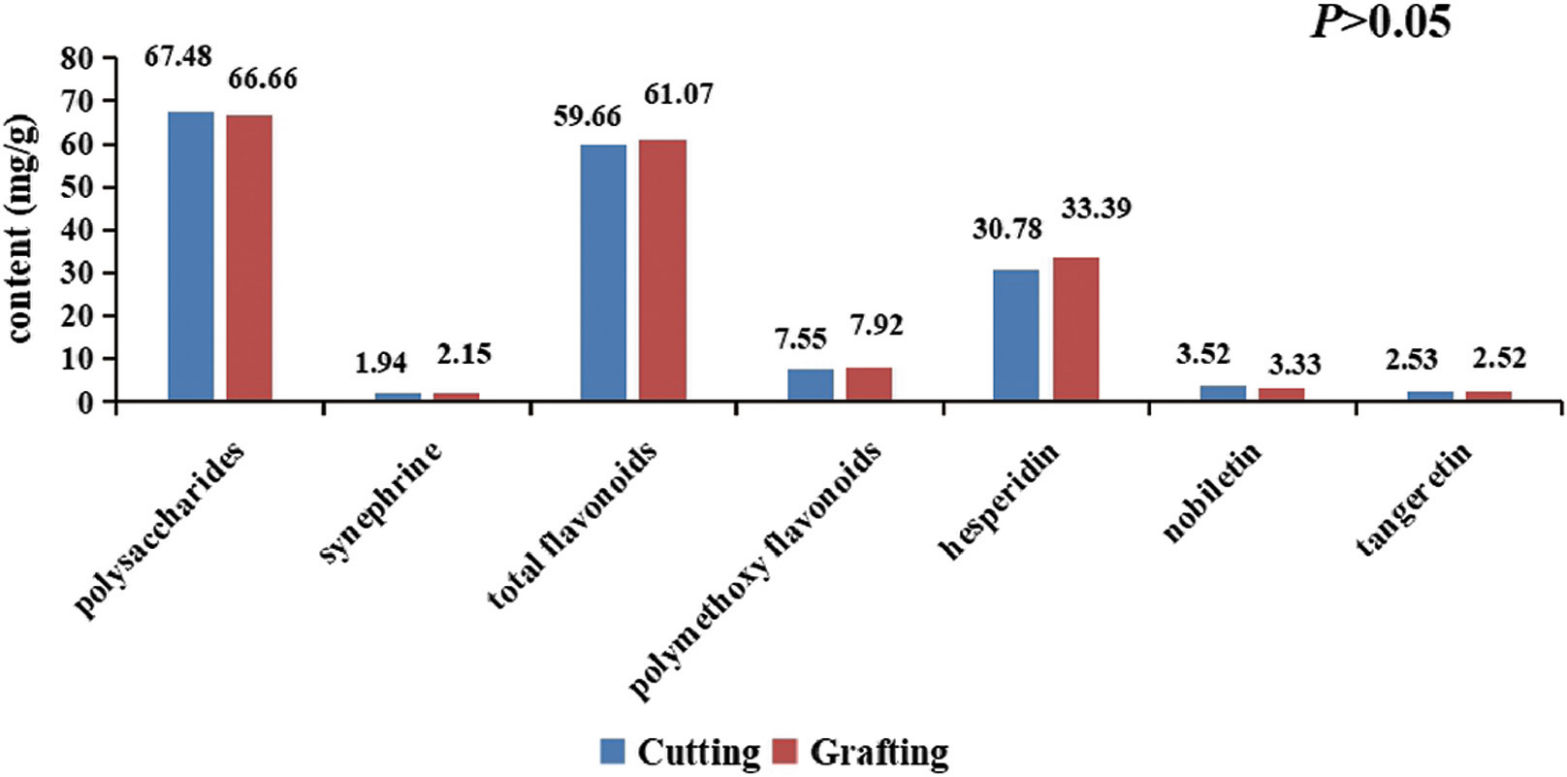
Column chart analysis of three metabolites in samples of cutting and grafting techniques

There are two major categories of flavonoids in XHCP, the flavanones, such as hesperidin, which is the predominant flavonoid in XHCP, and the polymethoxy flavonoids, represented mainly by nobiletin and tangeretin. First, we used UV spectrophotometry to quantify total flavonoids and total polymethoxy flavonoids in XHCP obtained from cutting and grafted plants. We observed that the average content of total flavonoids and total polymethoxy flavonoids was higher in grafted XHCP (61.07 and 7.92 mg/g, respectively) than in cutting XHCP (59.66 and 7.55 mg/g, respectively) (Figure 2, Figure S2, Table S8). Subsequently, the content of three individual flavonoids (i.e., hesperidin, nobiletin, and tangeretin) was determined. Hesperidin was present in the highest quantities, with concentrations ranging from 20.14 to 57.21 and 25.60 to 48.09 mg/g in cutting and grafted XHCP, respectively (Table S8). For the other two flavonoids, levels in cutting XHCP were higher or similar to those in grafted XHCP (*p* > .05). In cutting XHCP, the content of nobiletin varied from 2.78 to 4.50 mg/g, while tangeret content ranged between 1.90 and 3.21 mg/g (Table S8). In grafted XHCP, the nobilet in content was in the range from 2.59 to 3.95 mg/g, while the content of tangeret was between 1.85 and 3.31 mg/g (Table S8).

The flavonoids in *Citrus* have been proven to have antioxidant properties (Liew et al., 2018; Yi et al., 2008; Zang, 2019). Therefore, we tested the antioxidant activity of XHCP prepared from the two types of plants. We found that the DPPH radical scavenging rates of total flavonoids in XHCP were 73.50% (cutting XHCP) and 71.14% (grafted XHCP), while the reducing capacity of FRAP was 634.01 μmol/L FeSO_4_ and 656.26 μmol/L FeSO_4_, respectively (Table S9).

In general, the results presented in this section showed no significant differences (*p* > .05) in the contents and antioxidant activity of flavonoids in XHCP prepared from cutting and grafted plants (Table 2).

### 3.5 OPLS‐DA analysis of multicomponents of XHCP obtained by cutting and grafting

**FIGURE 3:**
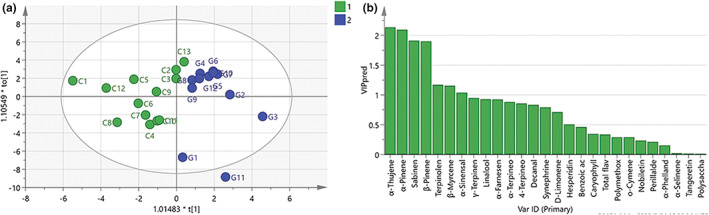
OPLS‐DA analysis of cutting XHCP and grafting XHCP. (a) Scores plot of OPLS‐DA model of 27 common components. (b) VIP value of 27 common components

The content of common components measured in 25 samples was used as a variable for OPLS‐DA analysis to obtain the corresponding model. The constructed OPLS‐DA model regarding the goodness of the fit and effect of predictive could be proven with R^2^_X_ > 0.5 (0.629) and Q^2^ > 0.4 (0.452). The OPLS‐DA score plot in Figure 3a shows a clear separation between the two groups even the intragroup dispersion of the two groups.

Potential marker metabolites were selected based on the criterion of variable Importance for the Projection (VIP) value greater than 1 and *p*‐value less than 0.05. Figure 3b shows that the chemical composition with VIP value >1 is α‐Thujene (2.13628), α‐Pinene (2.09823), Sabinen (1.91133), β‐Pinene (1.9014), Terpinolene (1.17065), β‐Myrcene (1.15528), and α‐Sinensal (1.0395), indicating that these components contribute greatly to the classification of cutting and grafting pericarp samples. Combining the aforementioned results (*p* < .05), α‐Thujene, α‐Pinene, Sabinen, β‐Pinene, and α‐Sinensal can be considered as potential chemical markers to distinguish the two groups.

## 4 DISCUSSION

In recent years, with the constant development of the citrus industry and the increasing awareness of functional foods (i.e., foods that are thought to have medicinal benefits), the total value of the XHCP industry has reached 10 billion CNY. However, alongside the rapid development of XHCP, the problem of Xinhui citrus planting methods has come to public attention. To explore this issue, we undertook a study of the various biochemical components of XHCP, on which its quality depends. XHCP contains a number of volatile components, flavonoids, alkaloids, carbohydrates, etc., and we focused our analysis on these. Flavonoids are one of the most active components in *Citrus*, and their antioxidant effects have been widely reported. Therefore, we also compared the antioxidant activity of total flavonoids in XHCP samples. According to different reaction principles, we used two different evaluation methods to obtain a more comprehensive understanding of the antioxidant activity.

We found that grafting onto *C. limonia* rootstock has no significant effect on the content of flavonoids, synephrine, total polysaccharides, and most essential oils, or antioxidant activity, in the pericarp of Xinhui citrus, which is in accord with the findings of Raddatz‐Mota et al. (2019), Machado et al. (2017), and Antonella et al. (2003). However, other researchers have expressed the view that rootstocks do have an impact on fruit quality (Aguilar‐Hernández et al., 2020; Cardeñosa et al., 2015; Feng et al., 2018; Morales et al., 2021; Tietel et al., 2020). These mixed results might be explained in several ways. First, the outcome may depend on which rootstock variety is used (Shen et al., 2020; Tietel et al., 2020; Wang, 2006). A few researchers have studied the effect on fruit quality of rootstocks of different genera. For instance, Wang (2006) compared the fruit quality of grafted and self‐rooted watermelon (*Citrullus lanatus*) and found no difference between self‐rooted plants and scions where rootstocks of the same genus (i.e., *Citrullus*) were used, but obtained the opposite result when self‐rooted plants were compared to watermelon grafted to rootstocks of a different genus (*Lagenaria siceraria*). Also important is the type of fruit structure analyzed (e.g., epicarp or endocarp) since the distribution of compounds is not homogeneous throughout the whole fruit. This point is particularly pertinent to those studies that mainly focused on compounds in fruit juice (Feng et al., 2018; Gil‐Izquierdo et al., 2004; Raddatz‐Mota et al., 2019), because other research has shown that flavonoids and volatile compounds in flavedo and albedo (epicarp and mesocarp) are present at higher levels than in juice sacs (epidermal hairs) (Ammar et al., 2015; Li et al., 2019; Wan, 2016).

In OPLS‐DA analysis, Q^2^ was greater than 0.4, indicating that the model we established has good prediction ability (Westerhuis et al., 2008; Worley & Powers, 2012), and can be used to identify cutting and grafting XHCP. Five potential chemical markers screened are associated with the scent of rosin, fruit, wood, and spice, in which the pharmacological effects of α‐Pinene and β‐Pinene are large as antibacterial agents and antioxidants (Liao et al., 2016; Salehi et al., 2019). However, in grafted XHCP, the content of α‐Thujene, α‐Pinene, Sabinen, and β‐Pinene was higher than that of cutting XHCP, which is contrary to the public's understanding of the better quality of cutting XHCP. In addition, the large within‐group variation in the scoring plot might be due to the lack of standardized cultivation of XHCP, as soil, tree age, fertilization, irrigation, pruning, and pest and disease control in cultivation might affect fruit quality (Cheng et al., 2015; Jiao et al., 2022; Khalid et al., 2016; Liu et al., 2021; Ma et al., 2022; Manthos & Rouskas, 2021).

The dispute over the quality difference between grafting XHCP and cutting XHCP is partly based on the obvious phenotypic variation between them. Phenotypic variation by grafting is generally caused by the interaction of rootstock, scion, and environment. And genetic experiments have demonstrated that all three genomes of a plant cell can engage in horizontal genome transfer between scion and rootstock (Bock, 2017; Fuentes et al., 2014; Hertle et al., 2021), which means that there will be genetic variation between plants grafted onto a rootstock and plants derived from cuttings. Nevertheless, *C*. *limonia* and *C*. *reticulate* “Chachi” are highly homologous *Citrus* species and analysis of four DNA barcodes (*rbc*L, *mat*K, *trn*H‐*psb*A, and ITS) showed a high degree of genetic stability (>99.81%) between plants grafted to *C*. *limonia* rootstock and *C*. *reticulata* “Chachi” cuttings (Liu et al., 2021). However, many genes in organelles that may influence agronomic characteristics (e.g., growth and stress tolerance) remain to be investigated. Hence, to gain insight into the effects of grafting on XHCP, genetic information exchange and the molecular mechanism of induced variation by grafting could be explored next.

## 5 CONCLUSIONS

Our study confirmed that the quality of grafting XHCP is not inferior to that of cutting XHCP, but rather increased the content of four of the five volatile constituents, which can also be considered as potential chemical markers to distinguish the two. This study will provide a theoretical basis for the scientific planting of XHCP.

## FUNDING INFORMATION

This work was supported by the Project of Administration of Traditional Chinese Medicine of Guangdong Province of China [grant numbers 20191368, 20222260, and 20223021] and Jiangmen City Basic and Applied Basic Research Foundation [grant number 2020[159]‐9].

## CONFLICT OF INTEREST

The authors declare that they have no known competing financial interests or personal relationships that could influence the work reported in this paper.

## Supporting information


**Table** **S1–S7** Supplementary Figures and Tables.

## Data Availability

Data availability statement;The data that support the findings of this study are available in the supplementary material of this article.
