# Supplementary material for: Comparative study on comprehensive quality of Xinhui chenpi by two main plant propagation techniques
Source: Food Sci Nutr. 2022 Nov 20;11(2):1104–12. doi: 10.1002/fsn3.3148 (PMC9922146; doi:10.1002/fsn3.3148)
Supplement: Supplementary file 1 — Table S1–S7 Supplementary Figures and Tables. [file FSN3-11-1104-s001.doc]

**Supplementary material**

Table S1. Calibration Curves of Six Chemical Compositions.

| Compound | Calibration Curve |
| --- | --- |
| total flavonoids | y=26.838x + 0.0077 (R² = 0.9996) |
| total polymethoxylated flavones | y=52.601x + 0.0009 (R² = 0.9997) |
| hesperidin | y=13235x + 329.66 (R² = 0.9997) |
| nobiletin | y=19740x + 11.796 (R² = 0.9995) |
| tangeretin | y=24542x - 6.8927 (R^2^=0.9998) |
| total polysaccharides | y = 62.897x - 0.0037 (R² = 0.9999) |
| synephrine | y = 24640x + 5.039 (R² = 0.9997) |

Table S2. Repeatability, Stability and Accuracy of Six Chemical Compositions.

| Compound | Repeatability RSD% | Stability RSD% | Accuracy |
| --- | --- | --- | --- |
| total flavonoids | 0.98% | 2.13% | 95.52% |
| total polymethoxylated flavones | 0.33% | 0.64% | 101.46% |
| hesperidin | 0.69% | 1.63% | 101.37% |
| nobiletin | 0.56% | 1.86% | 99.80% |
| tangeretin | 0.15% | 1.58% | 95.17% |
| total polysaccharides | 2.74% | 1.48% | 96.50% |
| synephrine | 1.37% | 1.58% | 104.02% |

Table S3. The volatile constituents of cutting Xinhui chenpi (n=3).

|  | Compound | C1 | C2 | C3 | C4 | C5 | C6 | C7 | C8 | C9 | C10 | C11 | C12 | C13 |  |
| --- | --- | --- | --- | --- | --- | --- | --- | --- | --- | --- | --- | --- | --- | --- | --- |
| 1 | α-Thujene | 0.16 | 0.51 | 0.57 | 0.33 | 0.42 | 0.38 | 0.36 | 0.22 | 0.43 | 0.45 | 0.43 | 0.25 | 0.45 |  |
| 2 | α-Pinene | 0.53 | 1.66 | 1.86 | 1.36 | 1.38 | 1.29 | 1.26 | 0.84 | 1.34 | 1.53 | 1.51 | 0.99 | 1.35 |  |
| 3 | Camphene | NA | NA | NA | NA | 0.01 | NA | NA | NA | NA | NA | NA | NA | NA |  |
| 4 | Sabinen | 0.12 | 0.22 | 0.17 | 0.15 | 0.20 | 0.20 | 0.20 | 0.13 | 0.19 | 0.21 | 0.20 | 0.11 | 0.25 |  |
| 5 | β-Pinene | 0.71 | 1.27 | 1.48 | 1.20 | 1.26 | 1.07 | 1.07 | 0.96 | 1.32 | 1.35 | 1.34 | 0.99 | 1.4 |  |
| 6 | β-Myrcene | 1.69 | 2.33 | 2.41 | 2.19 | 2.25 | 2.23 | 2.28 | 1.80 | 2.10 | 2.20 | 2.21 | 2.17 | 2.36 |  |
| 7 | Octanal | 0.03 | NA | 0.03 | NA | 0.06 | 0.06 | 0.06 | 0.04 | 0.08 | 0.08 | 0.07 | 0.05 | 0.05 |  |
| 8 | α-Phellandrene | 0.06 | 0.05 | 0.09 | 0.02 | 0.07 | 0.05 | 0.05 | 0.05 | 0.06 | 0.06 | 0.05 | 0.07 | 0.05 |  |
| 9 | α-Terpinene | 0.23 | 0.33 | 0.27 | 0.18 | NA | 0.23 | 0.27 | 0.15 | 0.27 | 0.22 | 0.20 | NA | 0.25 |  |
| 10 | o-Cymene | 2.30 | 2.38 | 3.23 | 4.27 | 2.41 | 2.45 | 2.46 | 3.91 | 3.72 | 3.47 | 3.91 | 3.85 | 3.79 |  |
| 11 | D-Limonene | 70.62 | 73.22 | 69.53 | 70.11 | 69.92 | 71.97 | 71.91 | 64.71 | 66.37 | 68.07 | 67.89 | 71.98 | 70.65 |  |
| 12 | γ-Terpinene | 17.22 | 15.14 | 16.13 | 13.82 | 16.86 | 14.6 | 14.18 | 16.42 | 17.81 | 15.67 | 14.6 | 15.2 | 16.15 |  |
| 13 | Terpinolene | 1.06 | 0.85 | 0.91 | 0.75 | 1.29 | 0.85 | 0.81 | 0.80 | 1.00 | 0.87 | 0.79 | 1.16 | 0.96 |  |
| 14 | Linalool | 0.06 | 0.06 | 0.06 | 0.16 | 0.07 | 0.13 | 0.18 | 0.23 | 0.20 | 0.19 | 0.19 | 0.07 | 0.09 |  |
| 15 | Nonanal | NA | NA | NA | NA | NA | 0.06 | NA | 0.06 | 0.10 | NA | 0.05 | 0.05 | 0.05 |  |
| 16 | cis-(S)-(-)-Limonene oxide | NA | NA | NA | 0.18 | NA | 0.08 | 0.09 | 0.33 | 0.17 | 0.25 | 0.24 | 0.06 | NA |  |
| 17 | Limonene oxide, trans- | NA | NA | NA | 0.16 | NA | 0.08 | 0.08 | 0.29 | 0.13 | 0.17 | 0.17 | NA | 0.04 |  |
| 18 | 3-Cyclohexene-1-carboxaldehyde | NA | NA | NA | 0.07 | NA | NA | NA | NA | 0.10 | NA | NA | NA | NA |  |
| 19 | Citronellal | 0.08 | NA | NA | NA | NA | NA | NA | NA | NA | NA | NA | NA | NA |  |
| 20 | (3E,5E)-2,6-Dimethylocta-3,5,7-trien-2-ol | NA | NA | NA | NA | NA | NA | NA | 0.07 | NA | NA | 0.04 | NA | NA |  |
| 21 | 4-Terpineol | 0.09 | 0.10 | 0.13 | 0.2 | 0.11 | 0.12 | 0.22 | 0.35 | 0.24 | 0.19 | 0.23 | 0.14 | 0.09 |  |
| 22 | p-Cymen-8-ol | NA | NA | NA | NA | NA | NA | NA | NA | 0.03 | NA | 0.04 | NA | NA |  |
| 23 | α-Terpineol | 0.12 | 0.16 | 0.20 | 0.43 | 0.25 | 0.30 | 0.47 | 0.73 | 0.48 | 0.47 | 0.55 | 0.20 | 0.10 |  |
| 24 | Decanal | 0.27 | 0.17 | 0.15 | 0.20 | 0.23 | 0.28 | 0.21 | 0.34 | 0.30 | 0.26 | 0.27 | 0.25 | 0.31 |  |
| 25 | cis-Carveol | NA | NA | NA | 0.04 | NA | NA | NA | 0.09 | NA | NA | NA | NA | NA |  |
| 26 | Citronellol | NA | 0.03 | NA | 0.03 | NA | NA | 0.09 | NA | NA | NA | NA | NA | NA |  |
| 27 | (-)-Carvone | NA | NA | NA | 0.04 | NA | 0.02 | 0.04 | 0.07 | 0.03 | NA | 0.04 | NA | NA |  |
| 28 | Citral | NA | NA | NA | 0.03 | NA | NA | NA | 0.09 | NA | NA | NA | NA | NA |  |
| 29 | Perillaldehyde | 0.04 | 0.04 | 0.04 | 0.07 | 0.05 | 0.07 | 0.12 | 0.14 | 0.08 | 0.07 | 0.07 | 0.03 | 0.02 |  |
| 30 | Thymol | 0.03 | 0.06 | 0.11 | 0.21 | 0.18 | 0.28 | 0.10 | 0.21 | 0.08 | 0.15 | 0.25 | 0.05 | NA |  |
| 31 | Carvacrol | NA | NA | 0.02 | NA | NA | NA | 0.07 | 0.22 | 0.04 | 0.05 | 0.07 | NA | NA |  |
| 32 | Acetic acid, trichloro-, nonyl ester | NA | NA | NA | NA | NA | NA | NA | NA | NA | NA | NA | 0.04 | 0.03 |  |
| 33 | 2-Cyclohexen-1-ol, 1-methyl-4-(1-methylethenyl)-, trans- | NA | NA | NA | NA | NA | NA | NA | 0.09 | NA | 0.05 | NA | NA | NA |  |
| 34 | Undecanal | 0.10 | NA | 0.02 | NA | NA | 0.04 | 0.09 | 0.06 | 0.03 | NA | 0.03 | NA | NA |  |
| 35 | Citronellol acetate | NA | NA | NA | NA | NA | NA | NA | 0.04 | NA | NA | NA | NA | NA |  |
| 36 | Copaene | 0.03 | 0.03 | 0.03 | 0.05 | 0.06 | 0.05 | 0.07 | 0.07 | 0.04 | 0.05 | 0.07 | 0.08 | 0.02 |  |
| 37 | 7-Tetradecenal, (Z)- | NA | NA | NA | NA | NA | NA | NA | NA | NA | NA | NA | 0.03 | NA |  |
| 38 | Benzoic acid, 2-(methylamino)-, methyl ester | 0.63 | 0.6 | 1.03 | 1.57 | 1.29 | 1.01 | 1.51 | 2.36 | 1.41 | 1.62 | 1.73 | 0.67 | 0.57 |  |
| 39 | Dodecanal | 0.12 | 0.05 | 0.08 | NA | 0.10 | NA | 0.08 | 0.16 | 0.13 | NA | NA | NA | NA |  |
| 40 | Caryophyllene | 0.11 | 0.10 | 0.16 | 0.19 | 0.18 | 0.2 | 0.15 | 0.15 | 0.12 | 0.16 | 0.17 | 0.17 | 0.11 |  |
| 41 | Humulene | 0.02 | NA | 0.08 | NA | NA | 0.03 | NA | NA | NA | NA | NA | NA | NA |  |
| 42 | 2-Dodecenal, (E)- | NA | NA | NA | NA | 0.04 | 0.03 | NA | NA | NA | 0.03 | NA | NA | NA |  |
| 43 | α-Selinene | 0.04 | 0.03 | 0.04 | 0.07 | 0.04 | 0.05 | 0.05 | 0.07 | 0.02 | 0.07 | 0.04 | 0.06 | 0.02 |  |
| 44 | α-Farnesene | 2.03 | 0.38 | 0.48 | 0.64 | 0.53 | 0.68 | 0.63 | 1.39 | 0.74 | 0.78 | 0.83 | 0.48 | 0.43 |  |
| 45 | δ-Cadinene | NA | NA | NA | NA | NA | NA | NA | 0.09 | 0.03 | NA | NA | NA | 0.03 |  |
| 46 | Caryophyllene oxide | NA | NA | NA | 0.05 | NA | NA | 0.02 | 0.08 | 0.03 | 0.04 | 0.06 | NA | NA |  |
| 47 | Octadecanal | NA | NA | NA | NA | NA | NA | NA | NA | NA | NA | 0.02 | NA | NA |  |
| 48 | α-Sinensal | 1.35 | 0.26 | 0.61 | 0.77 | 0.65 | 0.78 | 0.68 | 1.26 | 0.6 | 0.77 | 0.99 | 0.51 | 0.22 |  |
| 49 | β-Cadinene, (-)- | NA | NA | 0.04 | 0.06 | 0.08 | 0.07 | 0.08 | NA | NA | 0.07 | 0.08 | 0.06 | NA |  |
| 50 | 1,11-Hexadecadiyne | 0.05 | NA | NA | NA | NA | NA | NA | NA | NA | NA | NA | NA | NA |  |
| 51 | 3-Cyclohexen-1-ol, 5-methylene-6-(1-methylethenyl)-, acetate | NA | NA | NA | 0.07 | NA | NA | NA | NA | NA | NA | NA | NA | NA |  |
| 52 | Carveol | NA | NA | NA | 0.08 | NA | NA | NA | 0.08 | NA | NA | NA | NA | NA |  |
| 53 | 1,5,9,11-Tridecatetraene, 12-methyl-, (E,E)- | NA | NA | NA | 0.06 | NA | NA | NA | NA | NA | NA | NA | 0.02 | 0.04 |  |
| 54 | Tetradecanal | NA | NA | NA | 0.13 | NA | 0.12 | NA | NA | NA | 0.10 | 0.10 | 0.11 | 0.10 |  |
| 55 | Espatulenol | NA | NA | NA | 0.04 | NA | NA | NA | NA | NA | NA | 0.04 | NA | NA |  |
| 56 | 2-Pentyl-2-nonenal | NA | NA | NA | 0.05 | NA | NA | NA | 0.12 | NA | NA | 0.04 | NA | NA |  |
| 57 | (R)-(+)-Citronellal | NA | NA | NA | NA | NA | 0.06 | NA | NA | NA | NA | 0.03 | NA | NA |  |
| 58 | β-Elemene | NA | NA | NA | NA | NA | 0.05 | NA | NA | NA | NA | NA | NA | NA |  |
| 59 | Myrtenol | NA | NA | NA | NA | NA | NA | NA | 0.21 | NA | 0.12 | 0.12 | NA | NA |  |
| 60 | trans-p-mentha-1(7),8-dien-2-ol | NA | NA | NA | NA | NA | NA | NA | 0.16 | NA | NA | NA | NA | NA |  |
| 61 | (-)-trans-Isopiperitenol | NA | NA | NA | NA | NA | NA | NA | 0.07 | NA | NA | NA | NA | NA |  |
| 62 | cis-p-mentha-1(7),8-dien-2-ol | NA | NA | NA | NA | NA | NA | NA | 0.12 | NA | NA | NA | NA | NA |  |
| 63 | Linalool, formate | NA | NA | NA | NA | NA | NA | NA | 0.04 | NA | NA | NA | NA | NA |  |
| 64 | Heptadecanal | NA | NA | NA | NA | NA | NA | NA | 0.04 | NA | 0.04 | NA | NA | NA |  |
| 65 | cis-β-Ocimene | NA | NA | 0.03 | NA | NA | NA | NA | NA | 0.04 | NA | NA | NA | NA |  |
| 66 | Rhodinol | NA | NA | NA | NA | NA | NA | NA | NA | 0.06 | 0.06 | 0.07 | NA | NA |  |
| 67 | Cyclobutene, 4,4-dimethyl-1-(2,7-octadienyl)- | NA | NA | NA | NA | NA | NA | NA | NA | 0.03 | NA | NA | NA | NA |  |
| 68 | β-Cubebene | NA | NA | NA | NA | NA | NA | NA | NA | NA | 0.05 | NA | NA | NA |  |
| 69 | trans-Carveol | NA | NA | NA | NA | NA | NA | 0.03 | NA | NA | NA | 0.03 | NA | NA |  |
| 70 | α-trans-Bergamotenol | NA | NA | NA | NA | NA | NA | NA | 0.03 | NA | NA | 0.06 | NA | NA |  |
| 71 | Hydrazine | NA | NA | NA | NA | NA | NA | NA | NA | NA | NA | NA | 0.06 | NA |  |
| 72 | 2-Dodecenal | 0.03 | NA | 0.02 | NA | NA | NA | NA | NA | NA | NA | 0.02 | NA | 0.03 |  |
| 73 | trans-α-Bergamotene | NA | NA | NA | NA | NA | NA | NA | NA | 0.02 | NA | NA | NA | NA |  |
| 74 | Germacrene D | NA | NA | NA | NA | NA | NA | 0.03 | NA | NA | NA | NA | 0.04 | NA |  |
| 75 | trans-2-Tetradecenal | NA | NA | NA | NA | NA | NA | NA | NA | 0.02 | NA | NA | NA | NA |  |
| 76 | 2,6-Octadiene, 2,6-dimethyl- | NA | NA | NA | NA | NA | NA | NA | NA | NA | NA | 0.03 | NA | NA |  |
| 77 | Oxalic acid, octadecyl propyl ester | NA | NA | NA | NA | NA | NA | NA | NA | NA | 0.02 | NA | NA | NA |  |
| 78 | Decane, 2,3,5,8-tetramethyl- | NA | NA | NA | NA | NA | NA | NA | 0.03 | NA | NA | NA | NA | NA |  |
| 79 | α-Citral | NA | NA | NA | NA | NA | 0.03 | NA | NA | NA | NA | NA | NA | NA |  |
| 80 | 3-Ethyl-2,6,10-trimethylundecane | 0.03 | NA | NA | NA | NA | NA | NA | NA | NA | NA | NA | NA | NA |  |

NA: not available

Table S4. The RSD% of the results presented in Table S3.

|  | Compound | C1 | C2 | C3 | C4 | C5 | C6 | C7 | C8 | C9 | C10 | C11 | C12 | C13 |  |
| --- | --- | --- | --- | --- | --- | --- | --- | --- | --- | --- | --- | --- | --- | --- | --- |
| 1 | α-Thujene | 0.62 | 0.68 | 0.58 | 0.92 | 0.54 | 0.82 | 0.87 | 0.90 | 0.74 | 1.02 | 0.71 | 0.83 | 0.77 |  |
| 2 | α-Pinene | 0.98 | 0.55 | 0.48 | 0.62 | 0.51 | 0.60 | 0.71 | 0.64 | 0.57 | 0.66 | 0.83 | 0.59 | 0.74 |  |
| 3 | Camphene | NA | NA | NA | NA | 1.85 | NA | NA | NA | NA | NA | NA | NA | NA |  |
| 4 | Sabinen | 1.26 | 1.21 | 1.34 | 1.27 | 1.24 | 1.30 | 1.22 | 1.26 | 1.36 | 1.25 | 1.44 | 1.51 | 1.63 |  |
| 5 | β-Pinene | 0.94 | 0.84 | 0.84 | 0.80 | 0.79 | 0.95 | 0.84 | 1.03 | 0.85 | 0.96 | 0.74 | 0.88 | 0.65 |  |
| 6 | β-Myrcene | 0.75 | 0.66 | 0.73 | 0.70 | 0.84 | 0.65 | 0.60 | 0.71 | 0.65 | 0.68 | 0.68 | 0.77 | 0.74 |  |
| 7 | Octanal | 1.84 | NA | 1.81 | NA | 1.50 | 1.57 | 1.46 | 1.44 | 1.35 | 1.48 | 1.45 | 1.62 | 1.52 |  |
| 8 | α-Phellandrene | 1.70 | 1.82 | 1.64 | 1.57 | 1.38 | 1.44 | 1.70 | 1.76 | 1.63 | 1.55 | 1.67 | 1.48 | 1.50 |  |
| 9 | α-Terpinene | 1.37 | 1.08 | 1.11 | 1.17 | NA | 1.35 | 1.60 | 1.63 | 1.58 | 1.50 | 1.48 | NA | 1.77 |  |
| 10 | o-Cymene | 0.66 | 0.64 | 0.54 | 0.58 | 0.62 | 0.62 | 0.57 | 0.60 | 0.64 | 0.60 | 0.55 | 0.61 | 0.69 |  |
| 11 | D-Limonene | 0.70 | 0.63 | 0.68 | 0.75 | 0.75 | 0.58 | 0.66 | 0.70 | 0.69 | 0.42 | 0.47 | 0.58 | 0.50 |  |
| 12 | γ-Terpinene | 0.68 | 0.80 | 0.77 | 0.74 | 0.66 | 0.60 | 0.67 | 0.62 | 0.59 | 0.55 | 0.63 | 0.70 | 0.73 |  |
| 13 | Terpinolene | 0.96 | 0.83 | 0.85 | 0.92 | 0.91 | 0.76 | 0.72 | 0.85 | 0.70 | 0.75 | 0.88 | 0.85 | 0.75 |  |
| 14 | Linalool | 1.14 | 1.06 | 1.06 | 1.37 | 1.30 | 1.37 | 1.10 | 1.23 | 1.35 | 1.20 | 1.34 | 1.22 | 1.08 |  |
| 15 | Nonanal | NA | NA | NA | NA | NA | 1.02 | NA | 1.07 | 1.26 | NA | 1.01 | 0.92 | 1.16 |  |
| 16 | cis-(S)-(-)-limonene oxide | NA | NA | NA | 1.03 | NA | 1.14 | 1.22 | 1.17 | 1.08 | 1.05 | 1.06 | 1.32 | NA |  |
| 17 | Limonene oxide, trans- | NA | NA | NA | 1.16 | NA | 1.33 | 1.30 | 1.04 | 1.26 | 1.08 | 1.25 | NA | 1.24 |  |
| 18 | 3-Cyclohexene-1-carboxaldehyde | NA | NA | NA | 1.19 | NA | NA | NA | NA | 1.07 | NA | NA | NA | NA |  |
| 19 | Citronellal | 1.28 | NA | NA | NA | NA | NA | NA | NA | NA | NA | NA | NA | NA |  |
| 20 | (3E,5E)-2,6-Dimethylocta-3,5,7-trien-2-ol | NA | NA | NA | NA | NA | NA | NA | 1.56 | NA | NA | 1.47 | NA | NA |  |
| 21 | 4-Terpineol | 1.21 | 1.34 | 1.28 | 1.14 | 1.08 | 1.48 | 1.05 | 1.16 | 1.22 | 1.27 | 1.09 | 1.34 | 1.57 |  |
| 22 | p-Cymen-8-ol | NA | NA | NA | NA | NA | NA | NA | NA | 1.64 | NA | 1.43 | NA | NA |  |
| 23 | α-Terpineol | 1.14 | 1.09 | 1.18 | 1.20 | 0.97 | 1.19 | 0.85 | 1.29 | 1.17 | 1.11 | 1.09 | 1.13 | 0.98 |  |
| 24 | Decanal | 1.05 | 1.16 | 1.08 | 1.21 | 1.05 | 1.08 | 1.23 | 1.18 | 1.08 | 1.30 | 1.24 | 1.02 | 1.20 |  |
| 25 | cis-Carveol | NA | NA | NA | 1.36 | NA | NA | NA | 1.27 | NA | NA | NA | NA | NA |  |
| 26 | Citronellol | NA | 1.39 | NA | 1.42 | NA | NA | 1.49 | NA | NA | NA | NA | NA | NA |  |
| 27 | (-)-Carvone | NA | NA | NA | 1.08 | NA | 1.36 | 1.45 | 1.26 | 1.33 | NA | 1.22 | NA | NA |  |
| 28 | Citral | NA | NA | NA | 1.31 | NA | NA | NA | 1.22 | NA | NA | NA | NA | NA |  |
| 29 | Perillaldehyde | 1.45 | 1.50 | 1.32 | 1.63 | 1.52 | 1.61 | 1.50 | 1.73 | 1.42 | 1.34 | 1.27 | 1.36 | 1.47 |  |
| 30 | Thymol | 1.38 | 1.26 | 1.10 | 1.06 | 0.97 | 1.07 | 1.30 | 1.24 | 1.33 | 1.40 | 1.01 | 1.19 | NA |  |
| 31 | Carvacrol | NA | NA | 0.87 | NA | NA | NA | 0.93 | 1.35 | 1.42 | 1.21 | 1.26 | NA | NA |  |
| 32 | Acetic acid, trichloro-, nonyl ester | NA | NA | NA | NA | NA | NA | NA | NA | NA | NA | NA | 1.24 | 1.20 |  |
| 33 | 2-Cyclohexen-1-ol, 1-methyl-4-(1-methylethenyl)-, trans- | NA | NA | NA | NA | NA | NA | NA | 0.97 | NA | 0.99 | NA | NA | NA |  |
| 34 | Undecanal | 1.01 | NA | 1.20 | NA | NA | 1.14 | 1.25 | 1.17 | 1.10 | NA | 1.14 | NA | NA |  |
| 35 | Citronellol acetate | NA | NA | NA | NA | NA | NA | NA | 0.04 | NA | NA | NA | NA | NA |  |
| 36 | Copaene | 1.10 | 1.17 | 1.24 | 1.26 | 1.12 | 1.09 | 1.26 | 1.24 | 1.45 | 1.23 | 1.08 | 1.37 | 1.43 |  |
| 37 | 7-Tetradecenal, (Z)- | NA | NA | NA | NA | NA | NA | NA | NA | NA | NA | NA | 0.03 | NA |  |
| 38 | Benzoic acid, 2-(methylamino)-, methyl ester | 0.87 | 0.94 | 0.76 | 0.82 | 0.74 | 0.66 | 0.65 | 0.60 | 0.62 | 0.77 | 0.85 | 0.78 | 0.71 |  |
| 39 | Dodecanal | 1.12 | 1.40 | 1.10 | NA | 1.54 | NA | 1.42 | 1.36 | 1.05 | NA | NA | NA | NA |  |
| 40 | Caryophyllene | 0.95 | 1.33 | 0.96 | 0.84 | 1.25 | 1.34 | 0.84 | 0.79 | 1.18 | 1.26 | 1.31 | 1.29 | 1.32 |  |
| 41 | Humulene | 1.31 | NA | 1.27 | NA | NA | 1.29 | NA | NA | NA | NA | NA | NA | NA |  |
| 42 | 2-Dodecenal, (E)- | NA | NA | NA | NA | 1.45 | 1.40 | NA | NA | NA | 1.32 | NA | NA | NA |  |
| 43 | α-Selinene | 1.40 | 1.33 | 1.20 | 1.46 | 1.37 | 1.22 | 1.38 | 1.23 | 1.27 | 1.31 | 1.08 | 1.42 | 0.99 |  |
| 44 | α-Farnesene | 0.68 | 1.02 | 0.97 | 0.86 | 0.74 | 1.12 | 1.05 | 0.75 | 1.06 | 0.78 | 0.82 | 0.77 | 0.93 |  |
| 45 | δ-Cadinene | NA | NA | NA | NA | NA | NA | NA | 1.08 | 1.11 | NA | NA | NA | 1.14 |  |
| 46 | Caryophyllene oxide | NA | NA | NA | 1.13 | NA | NA | 1.21 | 1.08 | 1.36 | 1.10 | 1.17 | NA | NA |  |
| 47 | Octadecanal | NA | NA | NA | NA | NA | NA | NA | NA | NA | NA | 1.10 | NA | NA |  |
| 48 | α-Sinensal | 0.80 | 0.81 | 0.85 | 0.91 | 0.78 | 0.80 | 0.76 | 0.80 | 0.84 | 0.82 | 0.75 | 0.79 | 0.74 |  |
| 49 | β-Cadinene, (-)- | NA | NA | 1.54 | 1.32 | 1.40 | 1.37 | 1.24 | NA | NA | 1.30 | 1.30 | 1.25 | NA |  |
| 50 | 1,11-Hexadecadiyne | 1.03 | NA | NA | NA | NA | NA | NA | NA | NA | NA | NA | NA | NA |  |
| 51 | 3-Cyclohexen-1-ol, 5-methylene-6-(1-methylethenyl)-, acetate | NA | NA | NA | 0.97 | NA | NA | NA | NA | NA | NA | NA | NA | NA |  |
| 52 | Carveol | NA | NA | NA | 0.92 | NA | NA | NA | 0.80 | NA | NA | NA | NA | NA |  |
| 53 | 1,5,9,11-Tridecatetraene, 12-methyl-, (E,E)- | NA | NA | NA | 0.84 | NA | NA | NA | NA | NA | NA | NA | 1.15 | 1.17 |  |
| 54 | Tetradecanal | NA | NA | NA | 0.96 | NA | 0.82 | NA | NA | NA | 0.84 | 0.89 | 0.88 | 0.97 |  |
| 55 | Espatulenol | NA | NA | NA | 1.33 | NA | NA | NA | NA | NA | NA | 0.96 | NA | NA |  |
| 56 | 2-Pentyl-2-nonenal | NA | NA | NA | 1.26 | NA | NA | NA | 1.34 | NA | NA | 1.47 | NA | NA |  |
| 57 | (R)-(+)-Citronellal | NA | NA | NA | NA | NA | 1.39 | NA | NA | NA | NA | 1.12 | NA | NA |  |
| 58 | β-Elemene | NA | NA | NA | NA | NA | 1.42 | NA | NA | NA | NA | NA | NA | NA |  |
| 59 | Myrtenol | NA | NA | NA | NA | NA | NA | NA | 0.98 | NA | 1.02 | 1.10 | NA | NA |  |
| 60 | trans-p-mentha-1(7),8-dien-2-ol | NA | NA | NA | NA | NA | NA | NA | 0.83 | NA | NA | NA | NA | NA |  |
| 61 | (-)-trans-Isopiperitenol | NA | NA | NA | NA | NA | NA | NA | 0.97 | NA | NA | NA | NA | NA |  |
| 62 | cis-p-mentha-1(7),8-dien-2-ol | NA | NA | NA | NA | NA | NA | NA | 0.83 | NA | NA | NA | NA | NA |  |
| 63 | Linalool, formate | NA | NA | NA | NA | NA | NA | NA | 1.07 | NA | NA | NA | NA | NA |  |
| 64 | Heptadecanal | NA | NA | NA | NA | NA | NA | NA | 1.43 | NA | 1.50 | NA | NA | NA |  |
| 65 | cis-β-Ocimene | NA | NA | 1.47 | NA | NA | NA | NA | NA | 1.53 | NA | NA | NA | NA |  |
| 66 | Rhodinol | NA | NA | NA | NA | NA | NA | NA | NA | 1.09 | 1.14 | 1.00 | NA | NA |  |
| 67 | Cyclobutene, 4,4-dimethyl-1-(2,7-octadienyl)- | NA | NA | NA | NA | NA | NA | NA | NA | 1.57 | NA | NA | NA | NA |  |
| 68 | β-Cubebene | NA | NA | NA | NA | NA | NA | NA | NA | NA | 1.68 | NA | NA | NA |  |
| 69 | trans-Carveol | NA | NA | NA | NA | NA | NA | 1.71 | NA | NA | NA | 1.70 | NA | NA |  |
| 70 | α-trans-Bergamotenol | NA | NA | NA | NA | NA | NA | NA | 1.81 | NA | NA | 1.59 | NA | NA |  |
| 71 | Hydrazine | NA | NA | NA | NA | NA | NA | NA | NA | NA | NA | NA | 1.65 | NA |  |
| 72 | 2-Dodecenal | 1.65 | NA | 1.66 | NA | NA | NA | NA | NA | NA | NA | 1.80 | NA | 1.87 |  |
| 73 | trans-α-Bergamotene | NA | NA | NA | NA | NA | NA | NA | NA | 1.70 | NA | NA | NA | NA |  |
| 74 | Germacrene D | NA | NA | NA | NA | NA | NA | 1.54 | NA | NA | NA | NA | 1.48 | NA |  |
| 75 | trans-2-Tetradecenal | NA | NA | NA | NA | NA | NA | NA | NA | 1.63 | NA | NA | NA | NA |  |
| 76 | 2,6-Octadiene, 2,6-dimethyl- | NA | NA | NA | NA | NA | NA | NA | NA | NA | NA | 1.94 | NA | NA |  |
| 77 | Oxalic acid, octadecyl propyl ester | NA | NA | NA | NA | NA | NA | NA | NA | NA | 1.83 | NA | NA | NA |  |
| 78 | Decane, 2,3,5,8-tetramethyl- | NA | NA | NA | NA | NA | NA | NA | 1.60 | NA | NA | NA | NA | NA |  |
| 79 | α-Citral | NA | NA | NA | NA | NA | 1.42 | NA | NA | NA | NA | NA | NA | NA |  |
| 80 | 3-Ethyl-2,6,10-trimethylundecane | 1.59 | NA | NA | NA | NA | NA | NA | NA | NA | NA | NA | NA | NA |  |

NA: not available

Table S5. The volatile constituents of grafted Xinhui chenpi (n=3).

|  | Compound | G1 | G2 | G3 | G4 | G5 | G6 | G7 | G8 | G9 | G10 | G11 | G12 |  |
| --- | --- | --- | --- | --- | --- | --- | --- | --- | --- | --- | --- | --- | --- | --- |
| 1 | α-Thujene | 0.47 | 0.76 | 0.68 | 0.54 | 0.61 | 0.63 | 0.61 | 0.53 | 0.55 | 0.65 | 0.56 | 0.65 |  |
| 2 | α-Pinene | 1.99 | 3.11 | 2.93 | 1.75 | 1.99 | 1.95 | 1.94 | 1.66 | 1.78 | 2.05 | 2.49 | 2.12 |  |
| 3 | Camphene | NA | 0.02 | 0.02 | NA | NA | NA | NA | NA | NA | NA | 0.02 | NA |  |
| 4 | Sabinen | 0.17 | 0.21 | 0.31 | 0.25 | 0.22 | 0.25 | 0.22 | 0.23 | 0.23 | 0.26 | 0.24 | 0.22 |  |
| 5 | β-Pinene | 1.82 | 2.37 | 2.34 | 1.43 | 1.48 | 1.52 | 1.35 | 1.28 | 1.38 | 1.40 | 2.16 | 1.48 |  |
| 6 | β-Myrcene | 2.11 | 2.56 | 1.97 | 2.29 | 2.36 | 2.34 | 2.43 | 2.3 | 2.32 | 2.63 | 1.99 | 2.54 |  |
| 7 | Octanal | 0.11 | 0.05 | 0.34 | 0.05 | 0.07 | 0.05 | 0.09 | 0.08 | 0.04 | 0.1 | 0.17 | 0.08 |  |
| 8 | α-Phellandrene | 0.04 | 0.07 | 0.06 | 0.04 | 0.05 | 0.06 | 0.06 | 0.06 | 0.06 | 0.06 | 0.02 | 0.06 |  |
| 9 | α-Terpinene | 0.15 | 0.34 | 0.17 | 0.22 | 0.30 | 0.30 | 0.26 | 0.31 | 0.26 | 0.29 | 0.06 | 0.30 |  |
| 10 | o-Cymene | 5.88 | 2.71 | 3.46 | 3.43 | 2.70 | 3.03 | 2.50 | 1.95 | 2.72 | 2.22 | 8.09 | 2.68 |  |
| 11 | D-Limonene | 62.68 | 65.08 | 63.44 | 69.2 | 69.96 | 68.83 | 72.13 | 70.99 | 70.35 | 72.19 | 60.62 | 70.92 |  |
| 12 | Eucalyptol | NA | NA | 0.01 | NA | NA | NA | NA | NA | NA | NA | NA | NA |  |
| 13 | γ-Terpinene | 14.14 | 16.29 | 13.1 | 15.91 | 15.59 | 16.08 | 14.27 | 15.72 | 15.25 | 14.33 | 10.2 | 15.17 |  |
| 14 | Terpinolene | 0.77 | 0.98 | 0.71 | 0.82 | 0.88 | 0.87 | 0.79 | 0.85 | 0.80 | 0.83 | 0.62 | 0.85 |  |
| 15 | Linalool | 0.31 | 0.18 | 0.36 | 0.12 | 0.13 | 0.12 | 0.12 | 0.14 | 0.12 | 0.13 | 0.38 | 0.13 |  |
| 16 | Nonanal | 0.09 | 0.05 | 0.16 | NA | NA | NA | 0.04 | 0.04 | NA | 0.04 | 0.14 | 0.04 |  |
| 17 | cis-(S)-(-)-limonene oxide | 0.50 | 0.07 | 0.30 | 0.2 | 0.08 | 0.09 | 0.09 | 0.1 | 0.18 | NA | 0.39 | 0.13 |  |
| 18 | Limonene oxide, trans- | 0.34 | 0.07 | 0.30 | 0.16 | 0.06 | 0.08 | 0.07 | 0.08 | 0.13 | NA | 0.26 | 0.10 |  |
| 19 | 3-Cyclohexene-1-carboxaldehyde | 0.23 | NA | 0.27 | 0.13 | NA | 0.06 | 0.06 | 0.07 | 0.11 | NA | 0.16 | 0.08 |  |
| 20 | Citronellal | NA | NA | 0.15 | NA | NA | NA | NA | NA | NA | 0.05 | 0.06 | NA |  |
| 21 | (3E,5E)-2,6-Dimethylocta-3,5,7-trien-2-ol | 0.09 | NA | 0.09 | NA | NA | NA | NA | NA | NA | NA | 0.07 | NA |  |
| 22 | trans-L-Carvyl isobutyrate | NA | NA | 0.05 | NA | NA | NA | NA | NA | NA | NA | NA | NA |  |
| 23 | 4-Terpineol | 0.47 | 0.29 | 0.46 | 0.16 | 0.20 | 0.15 | 0.15 | 0.17 | 0.14 | 0.14 | 0.43 | 0.20 |  |
| 24 | p-Cymen-8-ol | 0.08 | NA | 0.06 | NA | NA | NA | NA | NA | NA | NA | 0.11 | NA |  |
| 25 | α-Terpineol | 0.86 | 0.45 | 1.02 | 0.35 | 0.44 | 0.36 | 0.30 | 0.36 | 0.33 | 0.31 | 0.84 | 0.38 |  |
| 26 | 1,5-Dimethyl-6-methylenespiro[2.4]heptane | NA | NA | 0.03 | NA | NA | NA | NA | NA | NA | NA | NA | NA |  |
| 27 | Decanal | 0.47 | 0.34 | 0.82 | 0.18 | 0.18 | 0.18 | 0.24 | 0.26 | 0.16 | 0.26 | 0.59 | 0.21 |  |
| 28 | cis-Carveol | 0.05 | NA | 0.05 | NA | NA | NA | NA | NA | NA | NA | 0.06 | NA |  |
| 29 | Nerol | NA | NA | 0.03 | NA | NA | NA | NA | NA | NA | NA | NA | NA |  |
| 30 | Citronellol | NA | NA | 0.14 | NA | NA | NA | NA | NA | NA | 0.05 | 0.14 | NA |  |
| 31 | (-)-Carvone | 0.05 | NA | 0.04 | NA | NA | NA | NA | NA | NA | NA | 0.07 | NA |  |
| 32 | Citral | NA | NA | 0.03 | NA | NA | NA | NA | NA | NA | NA | NA | NA |  |
| 33 | Perillaldehyde | 0.15 | 0.06 | 0.12 | 0.05 | 0.06 | 0.06 | 0.04 | 0.08 | 0.06 | 0.05 | 0.12 | 0.04 |  |
| 34 | 1,4-dihydroxy-p-menth-2-ene | NA | NA | 0.05 | NA | NA | NA | NA | NA | NA | NA | 0.04 | NA |  |
| 35 | Thymol | 0.24 | 0.09 | 0.02 | NA | 0.07 | NA | 0.03 | 0.1 | 0.13 | 0.04 | 0.22 | 0.05 |  |
| 36 | Nonyl trichloroacetate | NA | NA | 0.05 | NA | NA | NA | NA | NA | NA | NA | NA | NA |  |
| 37 | Carvacrol | 0.14 | 0.05 | 0.30 | 0.14 | 0.03 | 0.18 | 0.06 | 0.03 | 0.06 | NA | 0.06 | NA |  |
| 38 | Acetic acid, trichloro-, nonyl ester | 0.06 | NA | 0.06 | NA | NA | NA | NA | NA | NA | NA | NA | NA |  |
| 39 | 2-Cyclohexen-1-ol, 1-methyl-4-(1-methylethenyl)-, trans- | 0.05 | 0.02 | 0.04 | NA | NA | NA | NA | NA | NA | NA | 0.14 | NA |  |
| 40 | Undecanal | 0.05 | 0.03 | 0.07 | NA | NA | NA | NA | 0.02 | NA | 0.02 | 0.06 | NA |  |
| 41 | cis-p-Mentha-2,8-dien-1-ol | 0.06 | 0.02 | 0.06 | NA | NA | NA | NA | NA | NA | NA | 0.15 | NA |  |
| 42 | 4-(2,2-Dimethyl-6-methylenecyclohexyl)butanal | NA | NA | 0.02 | NA | NA | NA | NA | NA | NA | NA | 0.06 | NA |  |
| 43 | 1,2-Cyclohexanediol, 1-methyl-4-(1-methylethenyl)- | 0.07 | 0.03 | 0.07 | NA | NA | NA | NA | NA | NA | NA | 0.03 | NA |  |
| 44 | Citronellol acetate | 0.05 | 0.04 | 0.05 | NA | NA | NA | NA | NA | NA | NA | NA | NA |  |
| 45 | (2R,4R)-p-Mentha-6,8-diene, 2-hydroperoxide | 0.08 | NA | 0.07 | NA | NA | NA | NA | NA | NA | NA | 0.44 | NA |  |
| 46 | Copaene | 0.13 | 0.06 | 0.09 | 0.04 | 0.03 | 0.04 | 0.05 | 0.03 | NA | 0.04 | NA | NA |  |
| 47 | Nerol acetate | 0.02 | NA | 0.02 | NA | NA | NA | NA | NA | NA | NA | 0.04 | NA |  |
| 48 | 9-Hexadecyn-1-ol | NA | NA | 0.07 | NA | NA | NA | NA | NA | NA | NA | NA | NA |  |
| 49 | 7-Tetradecenal, (Z)- | NA | NA | 0.04 | NA | NA | NA | NA | NA | NA | NA | 0.04 | NA |  |
| 50 | Benzoic acid, 2-(methylamino)-, methyl ester | 2.10 | 1.48 | 2.36 | 1.20 | 1.26 | 1.33 | 0.91 | 1.06 | 1.32 | 0.70 | 2.01 | 0.81 |  |
| 51 | Dodecanal | 0.16 | 0.13 | 0.24 | 0.08 | 0.07 | NA | 0.08 | 0.09 | 0.07 | 0.09 | 0.20 | 0.06 |  |
| 52 | Caryophyllene | 0.25 | 0.20 | 0.12 | 0.11 | 0.09 | 0.13 | 0.10 | 0.10 | 0.11 | 0.08 | 0.26 | 0.06 |  |
| 53 | Humulene | 0.04 | 0.03 | 0.04 | NA | NA | NA | NA | NA | NA | NA | 0.06 | NA |  |
| 54 | 2-Dodecenal, (E)- | 0.03 | 0.03 | 0.07 | NA | NA | 0.03 | 0.03 | 0.03 | NA | 0.04 | 0.04 | 0.02 |  |
| 55 | α-Selinene | 0.13 | 0.05 | 0.03 | 0.04 | 0.02 | 0.03 | 0.02 | 0.03 | 0.05 | 0.02 | 0.19 | 0.02 |  |
| 56 | α-Farnesene | 1.16 | 0.86 | 0.57 | 0.58 | 0.50 | 0.49 | 0.36 | 0.62 | 0.66 | 0.46 | 1.51 | 0.23 |  |
| 57 | δ-Cadinene | NA | NA | 0.06 | NA | NA | NA | NA | NA | NA | NA | NA | 0.03 |  |
| 58 | α-Limonene diepoxide | 0.04 | NA | 0.05 | NA | NA | NA | NA | NA | NA | NA | 0.05 | NA |  |
| 59 | Caryophyllene oxide | 0.11 | NA | 0.06 | 0.04 | NA | NA | NA | NA | 0.03 | NA | 0.11 | NA |  |
| 60 | Octadecanal | 0.03 | 0.02 | 0.06 | NA | NA | NA | NA | NA | NA | NA | NA | NA |  |
| 61 | 8-Hexadecenal, 14-methyl-, (Z)- | NA | NA | 0.03 | NA | NA | NA | NA | NA | NA | NA | NA | NA |  |
| 62 | α-Sinensal | 0.61 | 0.56 | 1.20 | 0.47 | 0.49 | 0.54 | 0.51 | 0.54 | 0.55 | 0.34 | 0.16 | 0.28 |  |
| 63 | β-Cadinene, (-)- | 0.14 | 0.07 | NA | 0.04 | 0.04 | 0.05 | 0.05 | 0.03 | 0.03 | 0.04 | 0.13 | NA |  |
| 66 | Carveol | NA | NA | NA | NA | NA | NA | NA | NA | NA | NA | 0.11 | NA |  |
| 67 | Tetradecanal | NA | NA | NA | NA | NA | 0.08 | NA | NA | NA | NA | NA | NA |  |
| 68 | β-Elemene | NA | NA | NA | NA | NA | NA | NA | NA | NA | 0.03 | 0.09 | NA |  |
| 69 | Heptadecanal | NA | NA | NA | NA | NA | NA | NA | NA | NA | NA | 0.06 | NA |  |
| 70 | cis-β-Ocimene | NA | NA | NA | NA | NA | NA | NA | NA | NA | NA | NA | 0.02 |  |
| 71 | Rhodinol | 0.11 | 0.06 | NA | NA | 0.03 | 0.05 | 0.03 | 0.05 | 0.05 | NA | NA | 0.04 |  |
| 72 | trans-Carveol | NA | NA | NA | NA | NA | NA | NA | NA | NA | NA | 0.03 | NA |  |
| 73 | Santolina triene | 0.03 | NA | NA | NA | NA | NA | NA | NA | NA | NA | 0.06 | NA |  |
| 74 | 2-Methyl-2-(4-methyl-3-pentenyl)cyclopropanecarbaldehyde | 0.02 | NA | NA | NA | NA | NA | NA | NA | NA | NA | NA | NA |  |
| 75 | 6-(Hydroxymethyl)-1,4,4-trimethylbicyclo[3.1.0]hexan-2-ol | 0.02 | NA | NA | NA | NA | NA | NA | NA | NA | NA | NA | NA |  |
| 76 | trans-Ascaridol glycol | 0.02 | NA | NA | NA | NA | NA | NA | NA | NA | NA | NA | NA |  |
| 77 | trans-α-Bergamotene | 0.02 | NA | NA | NA | NA | NA | NA | NA | NA | NA | NA | NA |  |
| 78 | Lignoceryl alcohol | NA | 0.04 | NA | NA | NA | NA | NA | NA | NA | NA | NA | NA |  |
| 79 | Lavandulyl acetate | NA | 0.04 | NA | NA | NA | NA | NA | NA | NA | NA | NA | NA |  |
| 80 | Ambrettolide | NA | 0.07 | NA | NA | NA | NA | NA | NA | NA | NA | NA | NA |  |
| 81 | Bicyclo[10.1.0]tridec-1-ene | NA | NA | NA | NA | NA | 0.04 | NA | NA | NA | NA | NA | NA |  |
| 82 | Limonene oxide | NA | NA | NA | NA | NA | NA | NA | NA | NA | 0.04 | NA | NA |  |
| 83 | Germacrene D | NA | NA | NA | NA | NA | NA | NA | NA | NA | 0.02 | 0.02 | NA |  |
| 84 | 4-Acetyl-1-methylcyclohexene | NA | NA | NA | NA | NA | NA | NA | NA | NA | NA | 0.04 | NA |  |
| 85 | 1-Decanol | NA | NA | NA | NA | NA | NA | NA | NA | NA | NA | 0.14 | NA |  |
| 86 | 1,2-Benzenedicarboxylic acid, bis(2-methylpropyl) ester | NA | NA | NA | NA | NA | NA | NA | NA | NA | NA | 0.33 | NA |  |
| 87 | Hexadecanoic acid, methyl ester | NA | NA | NA | NA | NA | NA | NA | NA | NA | NA | 2.06 | NA |  |

NA: not available

Table S6. The RSD% of the results presented in Table S5

|  | Compound | G1 | G2 | G3 | G4 | G5 | G6 | G7 | G8 | G9 | G10 | G11 | G12 |
| --- | --- | --- | --- | --- | --- | --- | --- | --- | --- | --- | --- | --- | --- |
| 1 | α-Thujene | 1.22 | 1.10 | 1.35 | 1.02 | 1.15 | 1.36 | 1.27 | 1.24 | 1.18 | 1.07 | 1.15 | 1.29 |
| 2 | α-Pinene | 0.77 | 0.63 | 0.66 | 0.58 | 0.55 | 0.71 | 0.69 | 0.88 | 0.75 | 0.81 | 0.94 | 0.66 |
| 3 | Camphene | NA | 1.35 | 1.17 | NA | NA | NA | NA | NA | NA | NA | 1.50 | NA |
| 4 | Sabinen | 1.09 | 1.17 | 1.06 | 1.23 | 1.10 | 1.22 | 1.09 | 1.14 | 1.05 | 1.00 | 1.12 | 1.07 |
| 5 | β-Pinene | 0.62 | 0.75 | 0.83 | 0.95 | 0.88 | 1.02 | 1.27 | 0.98 | 0.90 | 0.86 | 0.86 | 0.94 |
| 6 | β-Myrcene | 0.73 | 0.66 | 0.73 | 0.70 | 0.84 | 0.78 | 0.74 | 0.77 | 0.81 | 0.75 | 0.75 | 0.77 |
| 7 | Octanal | 0.99 | 1.43 | 1.07 | 1.25 | 1.31 | 1.46 | 1.07 | 1.21 | 1.17 | 1.16 | 1.30 | 1.27 |
| 8 | α-Phellandrene | 1.45 | 1.17 | 1.09 | 1.37 | 1.40 | 1.25 | 1.18 | 1.03 | 1.38 | 1.23 | 1.44 | 1.52 |
| 9 | α-Terpinene | 1.05 | 1.25 | 1.12 | 1.07 | 1.09 | 0.95 | 1.17 | 1.22 | 0.99 | 1.00 | 1.12 | 1.10 |
| 10 | o-Cymene | 0.53 | 0.75 | 0.71 | 0.68 | 0.60 | 0.75 | 0.71 | 0.69 | 0.80 | 0.77 | 0.63 | 0.91 |
| 11 | D-Limonene | 0.44 | 0.53 | 0.38 | 0.51 | 0.40 | 0.35 | 0.32 | 0.57 | 0.61 | 0.55 | 0.47 | 0.59 |
| 12 | Eucalyptol | NA | NA | 1.26 | NA | NA | NA | NA | NA | NA | NA | NA | NA |
| 13 | γ-Terpinene | 0.68 | 0.72 | 0.74 | 0.86 | 0.86 | 0.92 | 0.63 | 0.82 | 0.80 | 0.52 | 0.87 | 0.65 |
| 14 | Terpinolene | 1.06 | 1.30 | 1.26 | 1.20 | 1.14 | 1.38 | 1.17 | 1.06 | 1.35 | 1.17 | 1.09 | 1.38 |
| 15 | Linalool | 1.24 | 1.15 | 1.08 | 1.24 | 1.20 | 0.99 | 1.55 | 1.20 | 1.13 | 1.35 | 1.14 | 1.32 |
| 16 | Nonanal | 1.19 | 1.63 | 1.14 | NA | NA | NA | 1.67 | 1.29 | NA | 1.66 | 1.25 | 1.48 |
| 17 | cis-(S)-(-)-limonene oxide | 1.45 | 1.71 | 1.05 | 1.33 | 1.34 | 1.46 | 1.40 | 1.16 | 1.40 | NA | 1.18 | 1.05 |
| 18 | Limonene oxide, trans- | 1.33 | 1.15 | 1.27 | 1.25 | 1.18 | 1.20 | 1.31 | 1.23 | 1.09 | NA | 1.05 | 1.22 |
| 19 | 3-Cyclohexene-1-carboxaldehyde | 1.26 | NA | 1.00 | 1.22 | NA | 1.43 | 1.26 | 1.15 | 1.20 | NA | 1.17 | 1.44 |
| 20 | Citronellal | NA | NA | 1.09 | NA | NA | NA | NA | NA | NA | 1.18 | 1.25 | NA |
| 21 | (3E,5E)-2,6-Dimethylocta-3,5,7-trien-2-ol | 1.57 | NA | 1.33 | NA | NA | NA | NA | NA | NA | NA | 1.40 | NA |
| 22 | trans-L-Carvyl isobutyrate | NA | NA | 0.74 | NA | NA | NA | NA | NA | NA | NA | NA | NA |
| 23 | 4-Terpineol | 1.08 | 1.22 | 1.06 | 1.10 | 1.14 | 0.96 | 1.18 | 1.05 | 1.14 | 1.17 | 1.35 | 1.30 |
| 24 | p-Cymen-8-ol | 1.23 | NA | 1.43 | NA | NA | NA | NA | NA | NA | NA | 1.15 | NA |
| 25 | α-Terpineol | 1.25 | 1.41 | 1.07 | 1.22 | 1.20 | 1.35 | 1.28 | 1.22 | 1.13 | 1.36 | 1.19 | 1.24 |
| 26 | 1,5-Dimethyl-6-methylenespiro[2.4]heptane | NA | NA | 1.32 | NA | NA | NA | NA | NA | NA | NA | NA | NA |
| 27 | Decanal | 1.23 | 1.28 | 1.11 | 1.22 | 1.17 | 1.20 | 1.08 | 1.00 | 1.28 | 1.26 | 1.15 | 1.30 |
| 28 | cis-Carveol | 1.57 | NA | 1.52 | NA | NA | NA | NA | NA | NA | NA | 1.45 | NA |
| 29 | Nerol | NA | NA | 1.40 | NA | NA | NA | NA | NA | NA | NA | NA | NA |
| 30 | Citronellol | NA | NA | 1.36 | NA | NA | NA | NA | NA | NA | 1.55 | 1.30 | NA |
| 31 | (-)-Carvone | 1.33 | NA | 1.27 | NA | NA | NA | NA | NA | NA | NA | 1.51 | NA |
| 32 | Citral | NA | NA | 1.28 | NA | NA | NA | NA | NA | NA | NA | NA | NA |
| 33 | Perillaldehyde | 1.05 | 1.40 | 1.33 | 1.41 | 1.45 | 1.29 | 1.37 | 1.50 | 1.23 | 1.62 | 1.15 | 1.22 |
| 34 | 1,4-dihydroxy-p-menth-2-ene | NA | NA | 1.60 | NA | NA | NA | NA | NA | NA | NA | 1.54 | NA |
| 35 | Thymol | 1.10 | 1.33 | 1.54 | NA | 1.49 | NA | 1.41 | 1.27 | 1.36 | 1.60 | 1.17 | 1.20 |
| 36 | Nonyl trichloroacetate | NA | NA | 1.39 | NA | NA | NA | NA | NA | NA | NA | NA | NA |
| 37 | Carvacrol | 1.08 | 1.46 | 1.22 | 1.30 | 1.49 | 1.25 | 1.54 | 1.50 | 1.29 | NA | 1.41 | NA |
| 38 | Acetic acid, trichloro-, nonyl ester | 1.42 | NA | 1.50 | NA | NA | NA | NA | NA | NA | NA | NA | NA |
| 39 | 2-Cyclohexen-1-ol, 1-methyl-4-(1-methylethenyl)-, trans- | 1.57 | 1.43 | 1.37 | NA | NA | NA | NA | NA | NA | NA | 1.28 | NA |
| 40 | Undecanal | 1.37 | 1.29 | 1.15 | NA | NA | NA | NA | 1.48 | NA | 1.43 | 1.39 | NA |
| 41 | cis-p-Mentha-2,8-dien-1-ol | 1.46 | 1.51 | 1.28 | NA | NA | NA | NA | NA | NA | NA | 1.52 | NA |
| 42 | 4-(2,2-Dimethyl-6-methylenecyclohexyl)butanal | NA | NA | 1.45 | NA | NA | NA | NA | NA | NA | NA | 1.24 | NA |
| 43 | 1,2-Cyclohexanediol, 1-methyl-4-(1-methylethenyl)- | 1.18 | 1.40 | 1.23 | NA | NA | NA | NA | NA | NA | NA | 1.37 | NA |
| 44 | Citronellol acetate | 1.31 | 1.25 | 1.27 | NA | NA | NA | NA | NA | NA | NA | NA | NA |
| 45 | (2R,4R)-p-Mentha-6,8-diene, 2-hydroperoxide | 1.22 | NA | 0.07 | NA | NA | NA | NA | NA | NA | NA | 1.30 | NA |
| 46 | Copaene | 1.12 | 1.45 | 1.43 | 1.29 | 1.24 | 1.40 | 1.28 | 1.20 | NA | 1.36 | NA | NA |
| 47 | Nerol acetate | 1.65 | NA | 1.24 | NA | NA | NA | NA | NA | NA | NA | 1.27 | NA |
| 48 | 9-Hexadecyn-1-ol | NA | NA | 1.23 | NA | NA | NA | NA | NA | NA | NA | NA | NA |
| 49 | 7-Tetradecenal, (Z)- | NA | NA | 1.08 | NA | NA | NA | NA | NA | NA | NA | 1.16 | NA |
| 50 | Benzoic acid, 2-(methylamino)-, methyl ester | 0.62 | 0.75 | 0.59 | 0.88 | 0.71 | 0.60 | 1.21 | 0.94 | 0.82 | 0.99 | 0.53 | 0.91 |
| 51 | Dodecanal | 1.00 | 0.87 | 0.96 | 1.14 | 1.20 | NA | 1.34 | 1.17 | 1.24 | 1.07 | 0.99 | 1.25 |
| 52 | Caryophyllene | 1.31 | 1.14 | 1.11 | 0.98 | 0.85 | 1.15 | 1.52 | 1.09 | 0.95 | 1.15 | 1.20 | 1.33 |
| 53 | Humulene | 1.22 | 1.04 | 1.14 | NA | NA | NA | NA | NA | NA | NA | 1.20 | NA |
| 54 | 2-Dodecenal, (E)- | 0.87 | 1.25 | 1.02 | NA | NA | 1.21 | 1.07 | 1.19 | NA | 1.06 | 1.27 | 1.41 |
| 55 | α-Selinene | 0.93 | 1.17 | 1.13 | 1.18 | 1.06 | 1.14 | 1.24 | 1.36 | 1.25 | 1.47 | 1.20 | 1.17 |
| 56 | α-Farnesene | 0.85 | 1.28 | 1.26 | 1.07 | 0.98 | 1.09 | 1.07 | 1.42 | 1.14 | 1.05 | 0.84 | 0.96 |
| 57 | δ-Cadinene | NA | NA | 1.20 | NA | NA | NA | NA | NA | NA | NA | NA | 1.15 |
| 58 | α-Limonene diepoxide | 1.20 | NA | 1.34 | NA | NA | NA | NA | NA | NA | NA | 1.42 | NA |
| 59 | Caryophyllene oxide | 1.11 | NA | 1.06 | 1.24 | NA | NA | NA | NA | 1.43 | NA | 0.11 | NA |
| 60 | Octadecanal | 1.25 | 1.09 | 1.18 | NA | NA | NA | NA | NA | NA | NA | NA | NA |
| 61 | 8-Hexadecenal, 14-methyl-, (Z)- | NA | NA | 1.33 | NA | NA | NA | NA | NA | NA | NA | NA | NA |
| 62 | α-Sinensal | 1.38 | 1.06 | 0.84 | 1.03 | 1.05 | 1.14 | 0.97 | 1.09 | 1.20 | 1.15 | 1.10 | 1.24 |
| 63 | β-Cadinene, (-)- | 1.21 | 1.10 | NA | 1.19 | 1.23 | 1.11 | 1.22 | 1.05 | 1.10 | 1.37 | 1.16 | NA |
| 66 | Carveol | NA | NA | NA | NA | NA | NA | NA | NA | NA | NA | 1.08 | NA |
| 67 | Tetradecanal | NA | NA | NA | NA | NA | 1.17 | NA | NA | NA | NA | NA | NA |
| 68 | β-Elemene | NA | NA | NA | NA | NA | NA | NA | NA | NA | 1.37 | 1.24 | NA |
| 69 | Heptadecanal | NA | NA | NA | NA | NA | NA | NA | NA | NA | NA | 1.12 | NA |
| 70 | cis-β-Ocimene | NA | NA | NA | NA | NA | NA | NA | NA | NA | NA | NA | 1.41 |
| 71 | Rhodinol | 1.16 | 1.05 | NA | NA | 1.24 | 1.18 | 1.06 | 1.42 | 1.11 | NA | NA | 1.30 |
| 72 | trans-Carveol | NA | NA | NA | NA | NA | NA | NA | NA | NA | NA | 1.09 | NA |
| 73 | Santolina triene | 1.35 | NA | NA | NA | NA | NA | NA | NA | NA | NA | 1.22 | NA |
| 74 | 2-Methyl-2-(4-methyl-3-pentenyl)cyclopropanecarbaldehyde | 1.41 | NA | NA | NA | NA | NA | NA | NA | NA | NA | NA | NA |
| 75 | 6-(Hydroxymethyl)-1,4,4-trimethylbicyclo[3.1.0]hexan-2-ol | 1.53 | NA | NA | NA | NA | NA | NA | NA | NA | NA | NA | NA |
| 76 | trans-Ascaridol glycol | 1.22 | NA | NA | NA | NA | NA | NA | NA | NA | NA | NA | NA |
| 77 | trans-α-Bergamotene | 1.13 | NA | NA | NA | NA | NA | NA | NA | NA | NA | NA | NA |
| 78 | Lignoceryl alcohol | NA | 1.27 | NA | NA | NA | NA | NA | NA | NA | NA | NA | NA |
| 79 | Lavandulyl acetate | NA | 1.03 | NA | NA | NA | NA | NA | NA | NA | NA | NA | NA |
| 80 | Ambrettolide | NA | 1.25 | NA | NA | NA | NA | NA | NA | NA | NA | NA | NA |
| 81 | Bicyclo[10.1.0]tridec-1-ene | NA | NA | NA | NA | NA | 1.18 | NA | NA | NA | NA | NA | NA |
| 82 | Limonene oxide | NA | NA | NA | NA | NA | NA | NA | NA | NA | 1.16 | NA | NA |
| 83 | Germacrene D | NA | NA | NA | NA | NA | NA | NA | NA | NA | 1.27 | 1.41 | NA |
| 84 | 4-Acetyl-1-methylcyclohexene | NA | NA | NA | NA | NA | NA | NA | NA | NA | NA | 1.33 | NA |
| 85 | 1-Decanol | NA | NA | NA | NA | NA | NA | NA | NA | NA | NA | 0.90 | NA |
| 86 | 1,2-Benzenedicarboxylic acid, bis(2-methylpropyl) ester | NA | NA | NA | NA | NA | NA | NA | NA | NA | NA | 1.02 | NA |
| 87 | Hexadecanoic acid, methyl ester | NA | NA | NA | NA | NA | NA | NA | NA | NA | NA | 0.74 | NA |

NA: not available

Table S7. The contents of total polysaccharides and synephrine (mg/g) (n=3).

| Sample | total polysaccharides | RSD% | synephrine | RSD% |
| --- | --- | --- | --- | --- |
| C1 | 63.58 | 1.67 | 1.68 | 0.98 |
| C2 | 67.70 | 1.44 | 1.65 | 0.82 |
| C3 | 76.46 | 1.62 | 2.28 | 0.71 |
| C4 | 67.35 | 1.53 | 2.73 | 0.76 |
| C5 | 72.57 | 1.37 | 1.54 | 0.87 |
| C6 | 66.72 | 1.52 | 1.73 | 0.93 |
| C7 | 58.13 | 1.60 | 1.76 | 0.95 |
| C8 | 56.45 | 1.22 | 2.20 | 0.70 |
| C9 | 50.16 | 1.49 | 1.85 | 1.15 |
| C10 | 97.79 | 1.11 | 2.31 | 1.09 |
| C11 | 81.48 | 1.70 | 2.11 | 0.87 |
| C12 | 73.33 | 1.65 | 1.75 | 1.16 |
| C13 | 47.82 | 1.23 | 1.88 | 1.48 |
| G1 | 91.77 | 1.61 | 2.41 | 0.61 |
| G2 | 55.28 | 1.50 | 1.94 | 1.18 |
| G3 | 78.88 | 1.48 | 1.88 | 1.24 |
| G4 | 54.86 | 1.53 | 2.00 | 0.87 |
| G5 | 60.89 | 1.48 | 2.26 | 0.66 |
| G6 | 56.09 | 1.37 | 2.30 | 0.75 |
| G7 | 67.20 | 1.80 | 2.21 | 1.37 |
| G8 | 65.55 | 1.63 | 2.22 | 0.89 |
| G9 | 67.52 | 1.55 | 2.22 | 1.30 |
| G10 | 72.46 | 1.40 | 1.72 | 1.29 |
| G11 | 72.74 | 1.52 | 2.41 | 0.95 |
| G12 | 56.61 | 1.48 | 2.18 | 1.16 |

Table S8. The content of flavonoids (mg/g) (n=3).

| Sample | Total flavonoids | RSD% | total polymethoxylated flavones | RSD% | hesperidin | RSD% | nobiletin | RSD% | tangeretin | RSD% |
| --- | --- | --- | --- | --- | --- | --- | --- | --- | --- | --- |
| C1 | 51.25 | 1.47 | 6.36 | 1.61 | 23.69 | 1.03 | 2.78 | 0.65 | 2.05 | 1.20 |
| C2 | 56.43 | 1.04 | 7.06 | 1.13 | 23.51 | 1.52 | 3.36 | 0.89 | 2.57 | 0.96 |
| C3 | 63.49 | 1.26 | 6.42 | 1.62 | 31.59 | 1.32 | 3.32 | 0.73 | 2.10 | 1.03 |
| C4 | 71.22 | 1.40 | 7.18 | 1.58 | 57.21 | 0.19 | 3.92 | 0.75 | 2.51 | 1.45 |
| C5 | 48.06 | 1.79 | 8.18 | 0.77 | 20.14 | 1.75 | 3.56 | 0.92 | 2.20 | 0.97 |
| C6 | 67.12 | 1.20 | 7.93 | 1.03 | 34.53 | 0.87 | 3.75 | 0.94 | 2.74 | 0.98 |
| C7 | 67.18 | 0.81 | 9.59 | 1.22 | 34.61 | 0.82 | 4.50 | 0.85 | 3.13 | 0.73 |
| C8 | 62.61 | 1.32 | 8.28 | 0.96 | 31.51 | 1.35 | 3.51 | 0.94 | 2.23 | 1.02 |
| C9 | 53.09 | 0.79 | 6.57 | 1.62 | 23.58 | 1.46 | 3.44 | 0.74 | 2.70 | 1.43 |
| C10 | 66.54 | 1.54 | 9.88 | 1.07 | 37.87 | 1.29 | 4.15 | 0.81 | 3.21 | 0.91 |
| C11 | 64.89 | 1.22 | 10.18 | 0.81 | 34.91 | 1.55 | 3.81 | 0.88 | 2.98 | 1.05 |
| C12 | 54.37 | 0.74 | 6.63 | 1.36 | 24.89 | 1.24 | 3.42 | 0.74 | 2.48 | 1.37 |
| C13 | 51.82 | 1.62 | 5.58 | 1.06 | 30.67 | 1.32 | 2.79 | 0.63 | 1.90 | 0.94 |
| G1 | 65.09 | 0.67 | 8.10 | 1.18 | 38.36 | 1.41 | 3.84 | 0.88 | 3.12 | 0.85 |
| G2 | 62.01 | 0.84 | 8.02 | 1.09 | 28.59 | 1.49 | 3.86 | 0.74 | 2.83 | 0.77 |
| G3 | 53.18 | 1.07 | 6.87 | 0.99 | 28.57 | 1.30 | 2.69 | 0.87 | 2.00 | 1.05 |
| G4 | 55.17 | 1.40 | 7.20 | 1.37 | 26.92 | 1.24 | 2.78 | 0.81 | 1.85 | 1.61 |
| G5 | 56.26 | 1.32 | 7.94 | 1.64 | 31.81 | 1.55 | 2.99 | 0.67 | 2.41 | 0.99 |
| G6 | 57.53 | 1.07 | 6.42 | 1.13 | 32.40 | 1.47 | 2.59 | 0.78 | 1.88 | 1.08 |
| G7 | 58.41 | 0.72 | 6.76 | 1.45 | 40.17 | 0.93 | 3.53 | 0.78 | 2.70 | 0.74 |
| G8 | 59.36 | 1.33 | 8.64 | 1.08 | 31.85 | 1.26 | 3.07 | 0.74 | 2.37 | 0.95 |
| G9 | 66.19 | 1.21 | 8.50 | 1.35 | 38.64 | 1.43 | 3.22 | 0.77 | 2.59 | 0.87 |
| G10 | 61.61 | 1.48 | 8.75 | 1.44 | 25.60 | 1.25 | 3.62 | 0.93 | 2.50 | 1.06 |
| G11 | 79.01 | 1.30 | 9.86 | 1.01 | 48.09 | 1.04 | 3.95 | 0.68 | 3.31 | 1.18 |
| G12 | 58.97 | 1.11 | 8.03 | 0.98 | 29.68 | 1.62 | 3.86 | 0.72 | 2.67 | 1.35 |

Table S9. Antioxidant activity in DPPH and FRAP Methods (n=3).

| Samples | DPPH % | RSD% | FRAP umol/L | RSD% |
| --- | --- | --- | --- | --- |
| C1 | 0.76 | 0.04 | 620.44 | 0.00 |
| C2 | 0.79 | 0.04 | 656.04 | 0.00 |
| C3 | 0.83 | 0.03 | 714.87 | 0.00 |
| C4 | 0.69 | 0.04 | 575.54 | 0.43 |
| C5 | 0.76 | 0.03 | 717.45 | 0.00 |
| C6 | 0.74 | 0.04 | 582.25 | 0.00 |
| C7 | 0.68 | 0.00 | 566.77 | 0.38 |
| C8 | 0.69 | 0.03 | 711.77 | 0.00 |
| C9 | 0.64 | 0.04 | 629.21 | 0.00 |
| C10 | 0.76 | 0.00 | 596.70 | 0.00 |
| C11 | 0.75 | 0.00 | 677.72 | 0.00 |
| C12 | 0.73 | 0.04 | 602.37 | 0.00 |
| C13 | 0.75 | 0.00 | 591.02 | 0.00 |
| G1 | 0.70 | 0.04 | 668.94 | 0.00 |
| G2 | 0.75 | 0.03 | 713.84 | 0.00 |
| G3 | 0.72 | 0.04 | 660.17 | 0.00 |
| G4 | 0.70 | 0.00 | 625.60 | 0.00 |
| G5 | 0.80 | 0.00 | 690.10 | 0.40 |
| G6 | 0.73 | 0.00 | 618.37 | 0.00 |
| G7 | 0.58 | 0.00 | 605.99 | 0.00 |
| G8 | 0.69 | 0.04 | 611.15 | 0.38 |
| G9 | 0.75 | 0.03 | 759.25 | 0.00 |
| G10 | 0.64 | 0.00 | 591.02 | 0.00 |
| G11 | 0.70 | 0.00 | 687.52 | 0.00 |
| G12 | 0.77 | 0.00 | 643.14 | 0.00 |


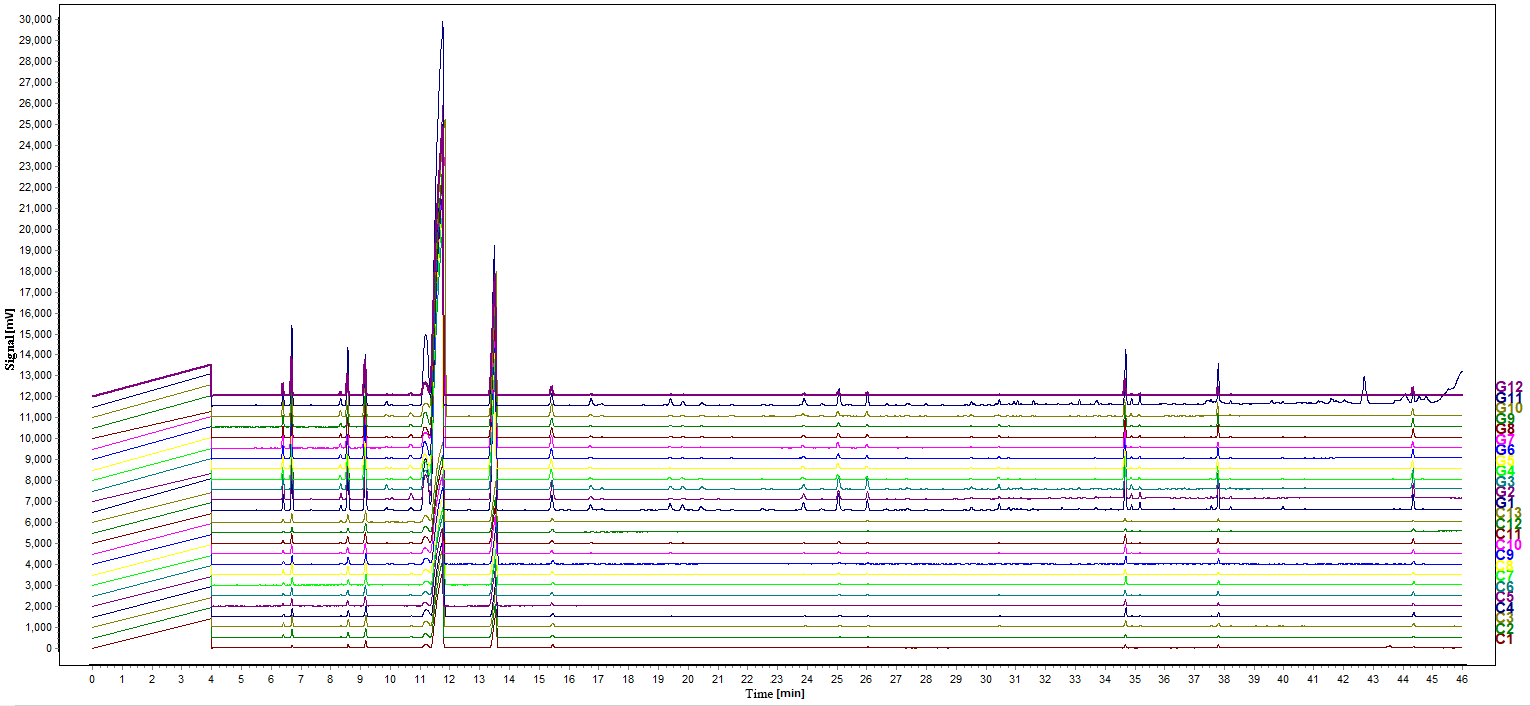


Fig. S1 The ion current chromatogram of volatile components of 25 samples.


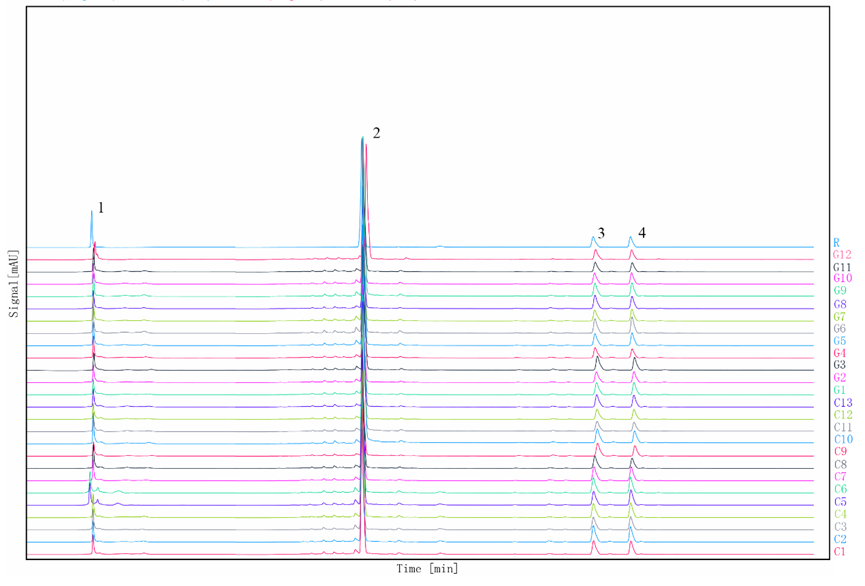


Fig. S2 HPLC chromatogram of reference materials (R) and 25 samples (1 for synephrine; 2 for hesperidin; 3 for nobiletin; 4 for tangeretin).
